# Pathways of Gastric Carcinogenesis, *Helicobacter pylori* Virulence and Interactions with Antioxidant Systems, Vitamin C and Phytochemicals

**DOI:** 10.3390/ijms21176451

**Published:** 2020-09-03

**Authors:** James W. T. Toh, Robert B. Wilson

**Affiliations:** 1 University of Sydney, UNSW, Westmead Hospital, Sydney, NSW 2145, Australia; james.toh@health.nsw.gov.au; 2 Department of Upper Gastrointestinal Surgery, UNSW, Liverpool Hospital, Liverpool, NSW 2170, Australia

**Keywords:** ascorbic acid, CagA, chronic atrophic gastritis, Correa pathway, dietary salt, gastric cancer, glutathione, *Helicobacter pylori*, nitrosamines, oxidative stress, phytochemicals, vitamin C

## Abstract

*Helicobacter pylori* is a class one carcinogen which causes chronic atrophic gastritis, gastric intestinal metaplasia, dysplasia and adenocarcinoma. The mechanisms by which *H. pylori* interacts with other risk and protective factors, particularly vitamin C in gastric carcinogenesis are complex. Gastric carcinogenesis includes metabolic, environmental, epigenetic, genomic, infective, inflammatory and oncogenic pathways. The molecular classification of gastric cancer subtypes has revolutionized the understanding of gastric carcinogenesis. This includes the tumour microenvironment, germline mutations, and the role of *Helicobacter pylori* bacteria, *Epstein Barr* virus and epigenetics in somatic mutations. There is evidence that ascorbic acid, phytochemicals and endogenous antioxidant systems can modify the risk of gastric cancer. Gastric juice ascorbate levels depend on dietary intake of ascorbic acid but can also be decreased by *H. pylori* infection, *H. pylori* CagA secretion, tobacco smoking, achlorhydria and chronic atrophic gastritis. Ascorbic acid may be protective against gastric cancer by its antioxidant effect in gastric cytoprotection, regenerating active vitamin E and glutathione, inhibiting endogenous N-nitrosation, reducing toxic effects of ingested nitrosodimethylamines and heterocyclic amines, and preventing *H. pylori* infection. The effectiveness of such cytoprotection is related to *H. pylori* strain virulence, particularly CagA expression. The role of vitamin C in epigenetic reprogramming in gastric cancer is still evolving. Other factors in conjunction with vitamin C also play a role in gastric carcinogenesis. Eradication of *H. pylori* may lead to recovery of vitamin C secretion by gastric epithelium and enable regression of premalignant gastric lesions, thereby interrupting the Correa cascade of gastric carcinogenesis.

## 1. Introduction

The Global Cancer statistics 2018 (GLOBOCAN 2018) estimated that in 2018 there were 1,033,701 new cases of gastric cancer in the world, with 782,685 deaths [1]. Behind lung cancer, this represented the second leading cause of cancer-related mortality worldwide, closely followed by hepatocellular/cholangio carcinoma [1,2,3,4,5]. There are marked global geographical variations in gastric cancer incidence, with the highest incidence in East Asian countries and the lowest incidence in North America and North Africa [1,2]. This is thought to be related to *Helicobacter pylori* prevalence and strain virulence, as well as additional gastric cancer risk factors [2,3,4].

Pathways for gastric cancer are complex and interrelated, including metabolic, environmental, epigenetic, genomic, infective, inflammatory and oncogenic. These involve risk factors such as *Helicobacter pylori* bacteria or *Epstein Barr* virus (EBV) infection, atrophic gastritis (AG), gastric intestinal metaplasia (IM), diet, high salt intake, tobacco smoking, alcohol consumption, obesity, racial background, ABO blood group, biological sex and family history of gastric cancer [6]. In 1994, the World Health Organization (WHO) and the International Agency for Research on Cancer classified *H. pylori* as a class 1 carcinogen [7].

This review provides an in-depth analysis of the molecular pathways to gastric cancer including the role of dietary and environmental risk factors—*H. pylori*, tobacco smoking, nitrosamines, heterocyclic amines, dietary salt and alcohol consumption amongst others. The inter-relationships between gastric cytoprotection, *H. pylori* virulence, epigenetics and gastric neoplasia are also examined. This includes the potential protective effect of endogenous antioxidant systems, vitamin C (ascorbic acid) and other ingested phytochemicals in the development of gastric cancer.

## 2. Gastric Carcinogenesis

### 2.1. Correa Pathway and Sydney Classification of Intestinal Metaplasia

Of the non-cardia gastric cancers, 89% are related to *H. pylori* bacterial infection and 9% are EBV related. The Correa model of intestinal type gastric carcinogenesis is a multistep cascade of chronic active gastritis progressing to multifocal AG, IM, low grade dysplasia, high grade dysplasia and finally gastric adenocarcinoma [8]. The Modified Sydney System is an internationally recognized endoscopic and histological system of chronic gastritis classification, which was originally introduced in 1994. It involves targeted endoscopic biopsies of abnormal gastric mucosa and non-targeted biopsies of the gastric antrum, incisura and corpus. This increases the detection rate of gastric IM, AG and dysplasia and can guide future endoscopic surveillance with mapping biopsies. The European Society of Gastrointestinal Endoscopy guideline currently includes eradication treatment for patients with *H. pylori*, and then endoscopic surveillance every three years in those found to have gastric IM [9].

Gastric intestinal metaplasia is classified as either focal or extensive in distribution, and complete or incomplete histological appearance. Complete gastric IM has small intestinal glands with loss of gastric mucins (MUC1, MUC5AC and MUC6), eosinophilic enterocytes with brush borders, well-defined goblet cells, and occasional Paneth cells. Incomplete gastric IM contains colonic type glands with intracytoplasmic mucin droplets and absence of an absorptive brush border. Incomplete IM has a higher proliferative rate and, together with extensive IM, is associated with a 4- to 11-fold higher risk for intestinal-type gastric cancer than complete IM [9,10,11].

Intestinal type gastric cancer and IM are characterized by high levels of Caudal type homeobox-2 (CDX-2) expression, which is also expressed in sporadic diffuse gastric cancer (DGC) but absent in normal gastric epithelium and in autosomal dominant hereditary gastric cancer (HDGC) [12,13]. DGC appears to arise directly from chronic active gastritis, without the intermediate steps of AG and IM in intestinal type gastric cancer. DGC is characterized by poorly cohesive adenocarcinoma cells with signet ring cell appearance on histopathology, resulting from intracellular mucin vacuoles which displace the nucleus to the periphery of the cell [10].

### 2.2. Helicobacter Pylori

**Figure 1:**
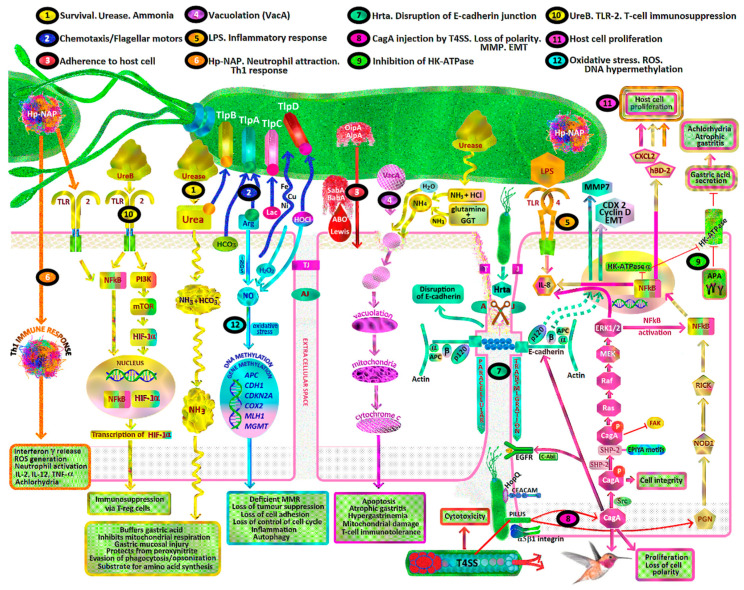
Pathways of *H. pylori* survival, chemotaxis, adhesion, colonization, virulence, inflammation, host immunotolerance, atrophic gastritis, oxidative stress, DNA methylation, cellular proliferation, EMT and oncogenesis in the gastric epithelium. *H. pylori* CagA injection causes disruption of E-cadherin and intercellular adhesion, loss of cell polarity, enhanced cell motility and development of the ‘hummingbird’ phenotype.

*Helicobacter pylori* is the first bacterial carcinogen described. It is usually acquired in childhood, with a long period of colonization and chronic gastritis eventually leading to non-cardia intestinal type gastric adenocarcinoma, sporadic diffuse gastric cancer or gastric B-cell lymphocyte mucosa associated lymphoid tissue (MALT) lymphoma [14]. Exposure to *H. pylori* toxins causes generation of gastric oxidative stress, reactive aldehyde formation, cellular DNA and RNA damage, hypermethylation of DNA promoter genes, host inflammatory response, chronic mucosal inflammation, achlorhydria, synergistic interactions with other carcinogens, and failure of antioxidant protection in the gastric mucosa. Such mechanisms involve:

1. *H. pylori* toxins which damage gastric mucosal epithelial cells leading to chronic atrophic gastritis, decreased gastric mucosal secretion of ascorbic acid, parietal cell apoptosis, achlorhydria, hypergastrinaemia, gastric dysbiosis, intestinal metaplasia, dysplasia and intestinal type gastric cancer (Figure 1, pathways 1, 4, 8, 9).
2. Recruitment of inflammatory cells resulting in acute and/or chronic inflammation, activation of reactive oxygen species (ROS) pathways including neutrophil myeloperoxidase- hypochlorite (HOCl)-hydrogen peroxide (H_2_O_2_), macrophage nitric oxide (NO) and epithelial cell hydrogen peroxide production (Figure 1, pathway 6, 12).
3. Oxidative stress due to reactive nitrogen species (RNS), ROS, lipid peroxidation and MDA/free radical formation overwhelming gastric antioxidant protection.
4. Promotion of gastric epithelial proliferation, oncogenes and DNA damage via various mechanisms, including *H. pylori* CagA, VacA, BabA, SabA, Hp-NAP, ROS, RNS, urease, DNA hypermethylation and cellular tyrosine kinases (Figure 1, pathways 1, 3, 4, 7, 8, 10, 11, 12).
5. Dysregulation of tyrosine kinase oncogenic pathways and loss of tumour suppressors (p53, CDH1/E-cadherin, APC, MGMT, MLH1, CDKN2A), leading to failure of apoptosis and Epithelial Mesenchymal Transition (EMT) (Figure 1, pathways 7, 8).
6. Synergy with ingested carcinogens (nitrosamines/heterocyclic amines/nitrites/dietary salt/alcohol/tobacco smoke) and complex interactions with antioxidants resulting in decreased protective effects and promotion of carcinogenesis (Figure 1).

### 2.3. Epidemiology of Gastric Cancer

**Figure 2:**
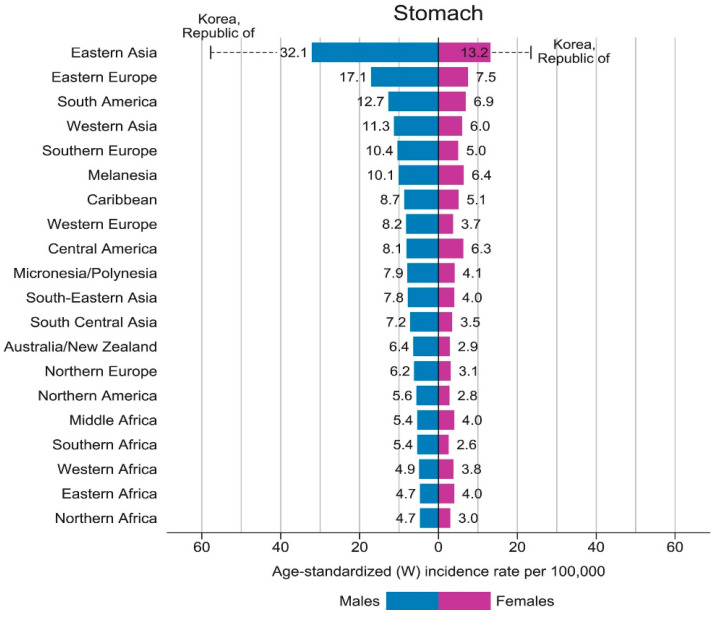
Age-standardized region-specific incidence (GLOBOCAN data) for gastric cancer in 2018. Adapted from Bray et al. (2018) with permission [1].

At least half of the world’s population is infected with *H. pylori*, but only 0.2–3% of those with *H. pylori* develop gastric cancer. From the GLOBOCAN 2018 data, the incidence of gastric adenocarcinoma is 3–12 times higher in East Asian countries (32.1/10^5^ males, 13.2/10^5^ females), Eastern Europe (17.1/10^5^ males, 7.5/10^5^ females) and Andean Latin America (26.9/10^5^ males, 10.3/10^5^ females) where virulent strains of *H. pylori* are endemic. This is compared to areas of lowest incidence in Australia/New Zealand (6.4/10^5^ males, 2.9/10^5^ females), North Africa (4.7/10^5^ males, 3.0/10^5^ females) and North America (5.6/10^5^ males, 2.8/10^5^ females). The highest rate of gastric cancer is in South Korea, with a national incidence of 60/10^5^ males and 25/10^5^ females [1] (Figure 2). The fall in incidence of non-cardia gastric cancer and peptic ulcer disease in Western developed countries has occurred in parallel with declines in the incidence of *H. pylori* colonization in these countries, particularly in persons under 65 years of age [15]. *H. pylori* is transferred by the oral-oral and faecal-oral routes, leading to intergenerational spread in families. The prevalence of *H. pylori* in developing countries is closely related to socio-economic status, as poor nutrition, overcrowding, inadequate sanitation and close personal contact increase colonization rates [16].

### 2.4. Lauren Classification

Since the Lauren classification was introduced in 1965, gastric cancer has been classified into intestinal, diffuse or mixed/indeterminate histological subtypes. Men are 2–4 times more likely to be diagnosed with intestinal-type non-cardia gastric cancer than females, whereas DGC is more common in women [14]. *H. pylori* is the main risk factor for non-cardia gastric adenocarcinoma and a diet rich in fresh fruits and vegetables is considered a protective factor [17,18]. Both diffuse and intestinal type gastric cancers share some dietary and environmental risk factors including *H. pylori* infection, as well as molecular abnormalities including DNA methylation, histone methylation and acetylation and chromosome recombination. The comparative risk of *H. pylori* causing intestinal type non-cardia gastric cancer (OR = 4.45; 95% CI: 2.74–7.24) versus DGC (OR = 3.39; 95% CI: 1.70–6.76) was equivalent (*p* = 0.50) in a pooled meta-analysis of 12 studies involving 1228 gastric cancer cases and 3406 controls [19,20].

Genetic predisposition is more common in HDGC, due to E-cadherin (*CDH1*) gene germline mutations in 40% of cases. HDGC makes up only 1–3% of overall gastric cancer cases. It is characterized by autosomal dominant inheritance, and is associated with a 70% lifetime risk of DGC [12,21]. The ‘two hit’ theory of gastric carcinogenesis suggests that loss of the second *CDH1* allele (by methylation, somatic mutations or loss of heterozygosity) is required for decreased E-cadherin glycoprotein expression and development of DGC. Sporadic DGC is associated with hypermethylation of the *CDH1* promoter gene in 35–55% of cases. Other gene mutations found in DGC include those of Ras homologue gene family, member A (RhoA), claudin-18 and Rho GTPase activating protein 6 (CLDN18-ARHGAP6), and *TGFβR1* [12,21].

Intestinal type non-cardia gastric cancers tend to affect male patients (M:F = 1.8:1), older patients (Male mean age = 50.4 years, Female mean age = 47.7 years) and metastasize via lymphatic and vascular invasion, with an associated longer clinical course and better prognosis than diffuse subtypes. Diffuse-type gastric cancers tend to arise in the gastric body, affect younger patients, particularly females, with a predilection for diffuse invasion of the gastric submucosa and muscularis propria and subsequent peritoneal metastasis. Blood group A is also associated with DGC [19,20].

### 2.5. Gastric Cancer Molecular Subtypes

In 2014, the Cancer Genome Atlas Research Network classified gastric carcinoma into four main molecular subtypes. These include:

1. *Epstein Barr* virus associated (EBV, 8.8%) with hypermethylation of DNA promoters. EBV gastric cancer is characteristically found in the proximal stomach. It is a poorly differentiated adenocarcinoma with lymphocytic infiltration on histology, PD-L1 and PD-L2 overexpression and CDKN2A silencing [22].
2. Microsatellite instability (MSI, 21.7%) due mainly to mutations of the hMLH1 gene promoter, leading to deficient mismatch repair of DNA (dMMR). MSI is associated with Lynch syndrome, distal gastric cancers and Lauren intestinal subtype on histology.
3. Chromosomal instability (CIN, 49.8%) with intestinal type cancer and cytosine and guanine (CpG) island methylator phenotype (CIMP). CIN gastric cancers arise more often in the gastro-oesophageal junction and gastric cardia (65%).
4. Genomically stable (GS, 19.7%) with DGC [23].

Subsequent transcriptomic and proteomic analysis has demonstrated that gastric cancer is a complex, heterogeneous disease, with substantial intra-tumoural, intra-patient and inter-patient variability [24,25,26]. The Lei classification (2013) is a biologically and therapeutically meaningful classification which divides gastric cancer into proliferative, metabolic and mesenchymal subtypes. In 2015, the Asian Cancer Research Group (ACRG) analysed the mRNA expression of 300 gastric cancers. Molecular subtypes were classified as:

1. MSI-high (23%),
2. Microsatellite stable/epithelial mesenchymal transition (MSS/EMT, 15%),
3. Microsatellite stable/TP53 intact (MSS/TP53+, p53 active, 26%)
4. Microsatellite stable/TP53 loss (MSS/TP53−, p53 inactive, 36%).
5. Each subtype was associated with distinct treatment options and prognostic outcomes [27].

### 2.6. Cardia vs. Non-Cardia Gastric Cancer

*H. pylori* appears not to be a significant risk factor for gastric cardia adenocarcinoma (OR = 0.99; 95% CI: 0.72–1.35) as compared to non-cardia adenocarcinoma (OR = 2.97; 95% CI: 2.34–3.77) [19]. Interactions between host susceptibility, genomics, environmental carcinogens, tissue protective mechanisms, redox status, and epigenetics appear to determine the progression of gastric carcinogenesis in both groups. However, infection with a virulent *H. pylori* strain (i.e., CagA+, VacA s1+) is the most important risk factor in non-cardia type gastric cancers [6].

### 2.7. Gastric Oxidative Stress

**Figure 3:**
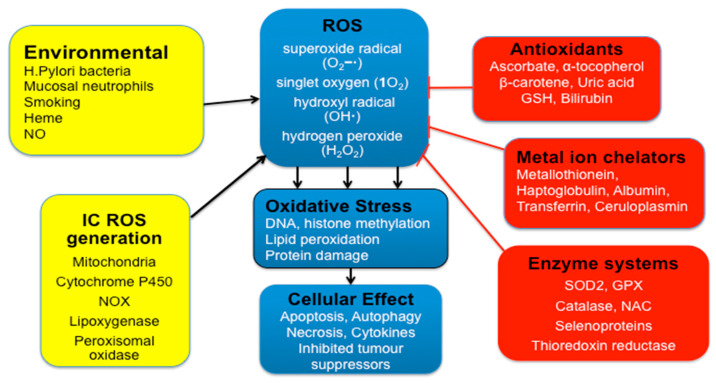
Contribution of environmental and intracellular (IC) sources of reactive oxygen species (ROS) with resulting oxidative stress and activation of oncogenes and inflammation. This can be modified by endogenous antioxidant systems, metal ion chelators and ingested phytochemicals including vitamin C (ascorbate) [30].

The stomach, as a bioreactor, is constantly exposed to ingested carcinogens and reactive species, bacterial pathogens and oxidative compounds related to food digestion [28]. Gastric cytoprotection and prevention of oxidative stress is dependent upon intact endogenous antioxidant systems and ingested antioxidants and phytochemicals in food [29]. ROS such as superoxide, hydrogen peroxide and peroxyl can steal electrons from cell membrane lipids, leading to lipid peroxidation and formation of unstable fatty acid radicals (lipid hydroperoxides) [30]. These break down to produce cytotoxic ketones, epoxides and reactive aldehydes such as glyoxal, methyl-glyoxal, acrolein, 4-hydroxynonenal and malondialdehyde (MDA) in the stomach. These are then absorbed in the intestines with a resulting rise in plasma and urinary levels. Reactive aldehydes damage protein and DNA, form DNA adducts such as malondialdehyde-deoxyguanosine, and also react with low density lipoproteins (LDL) to form MDA-LDL [29]. The generation of advanced lipid peroxidation end products (ALEs) and advanced glycation end products (AGEs) by reactive aldehydes (e.g., MDA) has been shown to be mutagenic in bacterial and mammalian systems and carcinogenic in rats [29,31] (Figure 3). The interaction of AGEs with their receptor (RAGE) promotes the progression of gastric cancer [32].

### 2.8. Gastric Cytoprotection

Gastric cytoprotection is reliant on antioxidant systems including:

1. Enzymatic antioxidants such as superoxide dismutase (SOD), catalase (CAT), thioredoxin reductase and glutathione peroxidase (GPX).
2. Small molecule antioxidants such as α-tocopherol (vitamin E), ascorbic acid (vitamin C), beta-carotene, bilirubin, glutathione (GSH) and uric acid.
3. Metal ion chelators including metallothionein, haptoglobulin, albumin, transferrin and ceruloplasmin (Figure 3).

These counteract ROS formation and free radical damage. Selenium is an important component of the selenoprotein antioxidant enzyme family, which includes glutathione peroxidases (GPX1–GPX4 and GPX6), thioredoxin reductases (TXNRD1–2), and thioredoxin-glutathione reductase (TXNRD3). The selenoproteins have the unique property of being able to rapidly remove hydrogen peroxide (H_2_O_2_) from the cell cytosol. This prevents H_2_O_2_ reacting with ferrous iron and forming hydroxyl radicals, which are the most reactive of oxygen derived free radicals. Selenium *deficiency* thus contributes to oxidative stress and enhanced inflammation [33].

Vitamin C is important in the reduction of oxidative stress at the gastric mucosal/luminal surface. It also maintains the antioxidant defence system by regenerating urate, glutathione, beta-carotene, and α-tocopherol (vitamin E). Glutathione can regenerate oxidised vitamin E (tocopheryl radical) or oxidised vitamin C (Dehydroascorbic acid; DHA), producing a thionyl radical (GS) in the process [34]. Vitamin C may act as a pro-oxidant at supraphysiological levels or in the presence of transition metal ions such as iron or copper, where, by Fenton chemistry, hydroxyl radicals are generated. For example, high dose oral ascorbic acid can have a *pro-oxidant* effect on ingested red meat (heme iron), resulting in lipid peroxidation in the stomach. However, when ascorbic acid is combined with polyphenols (e.g., catechins), they have synergistic antioxidant effects, and lipid peroxidation is prevented. Polyphenols contained in sage or rosemary can inhibit the formation of ALEs in the stomach from red meat by as much as 100%. One of the theories as to why individual oral antioxidant supplements may not be successful in large-scale human preventative studies is the widespread deficiency of dietary polyphenols or selenium. However, a diet rich in plant polyphenols *and* vitamin C, such as the Mediterranean diet, helps to maintain redox homeostasis in the stomach during a meal [29,35].

**Figure 4:**
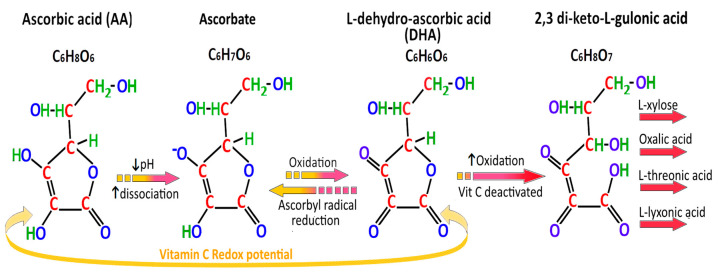
The redox potential of ascorbate. At the normal acidic gastric pH, secreted gastric ascorbic acid dissociates into ascorbate. Ascorbate can act as an antioxidant by reversible oxidation to DHA, and then be regenerated by glutathione-dependent DHA reductase back to ascorbate. DHA can be irreversibly oxidized to 2,3 di-keto-L-gulonic acid and thence excreted in the urine as oxalate.

The importance of fresh citrus fruit was demonstrated by the naval surgeon Dr. James Lind in 1747 on the HMS *Salisbury*, when he showed consumption of two oranges and a lemon per day cured scurvy in sailors [36]. Since then, the crucial role of vitamin C in collagen formation has been established, its deficiency leading to impaired hydroxylation of the amino acid proline to hydroxyproline by the Fe^2+^/2-oxoglutarate (2-OG)-dependent dioxygenase prolyl hydroxylase. This redox ability also makes vitamin C the major and essential water-soluble antioxidant in human serum and tissues. Vitamin C can function as an electron donor to form DHA, via the intermediate ascorbyl radical. Vitamin C can thus scavenge the superoxide anion radical (O_2_^−^**·**), singlet oxygen (1O_2_), hydroxyl radical (OH**·**), neutralize hypochlorous acid (HOCl), and prevent lipid peroxidation. Vitamin C cannot scavenge or neutralize hydrogen peroxide (H_2_O_2_). Rather, vitamin C may potentiate its toxicity by inhibiting catalase activity. HOCl is produced from H_2_O_2_ and Cl^−^ by myeloperoxidase (MPO) present in neutrophils [37,38] (Figure 3 and Figure 4).

Vitamin C can protect DNA from oxidant-mediated damage including CIMP and 8-hydroxy-2-deoxyguanosine (8-OHdG) formation. It has been reported to neutralize phagocyte-derived oxidants, protecting the 1-protease inhibitor (API) from oxidant-mediated functional inactivation. Vitamin C is also important in the antioxidant protection of lipid-soluble environments such as cell membranes, mitochondria, and endoplasmic reticulum by regeneration of vitamin E. It does so by donating an electron to the vitamin E radical to regenerate the active form of vitamin E, alpha-tocopherol [34]. The preventative effect of vitamin C on *H. pylori* generated malondialdehyde-deoxyguanosine is debated [29,31,39].

### 2.9. Absorption and Secretion of Ascorbic Acid

Humans, like other higher primates, fruit bats and guinea pigs do not have functional hepatic L-gulono-gamma-lactone oxidase (L-GULO), the enzyme which catalyzes the final step in the endogenous synthesis of ascorbic acid from glucose [40]. These particular mammals are thus at risk of scurvy (and oxidative stress) if oral vitamin C ingestion is inadequate. As such, dietary vitamin C intake, vitamin C transporters and levels of oxidative stress determine the plasma and organ levels of ascorbic acid. The US Food and Nutrition Board recommended daily allowance (RDA) of vitamin C in men is 90 mg and 75 mg in women. Normal plasma levels of vitamin C in humans are 30–90 μmol/L, with marginal deficiency at 11–23 μmol/L and deficiency at <11 μmol/L. Vitamin C is actively absorbed in the intestines via the two sodium-dependent vitamin C transporters (SVCT1 and SVCT2). DHA is absorbed via facilitated diffusion in the small intestines and also competes with glucose for active transport by enterocyte glucose transporters (GLUT2, GLUT8). Enterocytes (and also erythrocytes) contain GSH-dependent dehydroascorbate reductases which convert DHA to ascorbate, maintaining low intracellular DHA levels and a concentration gradient for continued DHA uptake [41]. The vitamin C renal threshold occurs above a plasma level of 80 μmol/L [41].

Vitamin C is actively secreted into the gastric lumen by gastric epithelial cells under the influence of gastrin, cholecystokinin (CCK) and acetyl choline [16,42]. Various mechanisms for vitamin C secretion have been proposed, including gap-junction hemi-channels, Ca^2+^-dependent anion channels, cell membrane homo- and hetero-exchange systems or exocytosis of ascorbate containing secretory vesicles [43].

Ascorbic acid levels are normally 3–10 times higher in gastric glands than in the plasma of human subjects. The accumulation of ascorbic acid in gastric glands occurs against an 8-fold concentration gradient by SVCT2, located in the basolateral membrane of the gastric epithelium. Analysis of ascorbic acid levels in KATO III and AGS gastric epithelial cells demonstrated a high affinity, saturable transport system for ascorbic acid with a K_m_ of 3–11 μmol/L. The concentration of gastric luminal ascorbate is decreased by *H. pylori* infection, poor oral vitamin C intake, use of proton pump inhibitors, cigarette smoking, impaired secretory state of the gastric mucosa, autoimmune gastritis, achlorhydria and atrophic gastritis. The average intake of oral vitamin C was found to be lower in *H. pylori* positive subjects (35.9 mg/day) as compared to 130.9 mg/day in *H. pylori* negative subjects (*p* < 0.01), which may also contribute to diminished intragastric ascorbate levels [41].

The oxidization of luminal gastric ascorbate to the reversible product DHA and the irreversible product 2,3-diketo-L-gulonic acid is related to the increased pH in *H. pylori*-associated corpus gastritis. In *H. pylori* positive patients with normal gastric pH of pH < 2, the mean gastric juice ascorbic acid level was 16.5 μmol/L, but fell to 4.5 μmol/L in those with gastric pH 2–4, and to zero in those with gastric pH > 6 [44]. Other mammals such as cats, rabbits, dogs, goats, rats and mice have preserved L-GULO function, and can markedly increase their vitamin C synthesis during oxidative stress [45,46] (Figure 4).

## 3. *Helicobacter pylori*-Virulence Factors and Pathways to Gastric Cancer

### 3.1. Helicobacter pylori

*H. pylori* is a microaerophilic, gram negative, flagellated, curved rod which infects half of the worldwide population. It has evolved to live in the acidic (luminal pH = 1.2–3.5) environment of the human stomach by its flagellar motility, chemotaxis signalling, urease activity, ammonia production, bacterial toxins and adhesin molecules. Once ingested, *H. pylori* bacteria penetrate the thick mucus layer (100 μm) of the stomach by swimming with their 4–8 unipolar flagella and digesting the viscous gastric mucus with ammonia. Their helical shape also enables penetration of the mucus layer by corkscrew-like, counterclockwise rotational movements. The ammonia is derived from *H. pylori* urease activity splitting urea into ammonia and CO_2_ gas, and *H. pylori* derived γ-glutamyl transpeptidase (GGT) hydrolyzing glutamine into ammonia [47].

### 3.2. H. pylori Urease

The *H. pylori* urease enzyme is initially intracytoplasmic, but, after gastric colonization commences, it can become surface associated. It may also be released into the extracellular space by secretion or by bacterial autolysis. The urease apoenzyme is assembled by a cytoplasmic histidine kinase, and is made up of two major subunits UreA and UreB. The dodecameric urease complex is then matured by the accessory proteins UreE/UreG and UreF/UreH, and activated by the insertion of two nickel ions (Ni^2+^). For host-derived urea to reach the urease in the bacterial cytoplasm, it has to be moved across both the *H. pylori* outer and inner membranes. This process is facilitated by a specific proton-gated urea pore in the inner membrane (UreI). The urea is hydrolyzed into two ammonia molecules (2NH_3_) and carbonic acid (H_2_CO_3_) by urease, and the carbonic acid is converted to CO_2_ gas by cytoplasmic *β*-carbonic anhydrase. UreI then enables movement of ammonia and CO_2_ back through the periplasm. An α-carbonic anhydrase located on the periplasm/inner membrane can generate HCO_3_^−^ from the returning CO_2_ for periplasmic buffering [48,49]. NH_4_^+^/NH3 (pKa = 9.3) and HCO_3_^−^/CO_2_ buffer gastric parietal cell derived HCl in the immediate external environment, but also in the bacterial cytoplasm and periplasm. By this mechanism, the *H. pylori* cytoplasmic pH is kept relatively neutral and periplasmic pH at around 6.1, even with an external pH (pH_e_) as low as 2.5. This maintains *H. pylori* intracellular homeostasis and permits successful bacterial colonization of the stomach. Transcription of *ureAB* is induced by the presence of acid and by nickel [50]. *H. pylori* urease activity increases exponentially as the external pH (pH_e_) decreases-the availability of urea at pH_e_ < 4 thus determines *H. pylori* survival in the stomach [51] (Figure 1, pathway 1). In conditions where host derived urea is limited, *H. pylori* arginase can endogenously produce urea and l-ornithine from l-arginine [52].

### 3.3. H. pylori-Derived Ammonia

Being a gas, ammonia readily diffuses across lipid membranes and reacts to form OH^−^ and NH_4_^+^ in the interior of the membrane. This raises the internal pH of intracellular organelles and can thus affect phagosome-lysosome fusion in mucosal macrophages and the function of epithelial cell mitochondria. The gastric juice of *H. pylori* positive subjects contains significantly higher concentrations of ammonia (0.01–0.02%) than that of *H. pylori* negative subjects (<0.005%). Such levels of ammonia in the gastric juice of *H. pylori* positive subjects are able to:

1. buffer gastric acid
2. inhibit gastric epithelial mitochondrial and isolated cellular respiration
3. induce cytotoxicity in gastric epithelial cells
4. contribute to gastric mucosal injury
5. enable *H. pylori* to evade host phagocytosis and opsonisation
6. provide protection to *H. pylori* from host generated peroxynitrite
7. provide a substrate for *H. pylori* from which to synthesise amino acids [47,53] (Figure 1, pathway 1).

### 3.4. H. pylori and Hypochlorhydria

Other mechanisms for the acute and profound hypochlorhydria which occurs within three days of initial *H. pylori* infection include:

1. Increased T helper type 1 (Th1) cell secretion of Interleukin-1β (from mucosal neutrophils and monocytes), which inhibits gastric proton pump (H^+^/K^+^-ATPase) activity (Figure 1, pathway 6).
2. Release of *H. pylori* fatty acids (tetradecanoic acid and cis 9,10-methyleneoctadecanoic acid), which inhibit proton pump H^+^/K^+^-ATPase and dissipate proton (H^+^) transport in parietal cell secretory vesicles [54].
3. *H. pylori* cag pathogenicity island (PAI) gene products repress the nuclear transcription of the catalytic alpha subunit of the parietal cell proton pump (HKalpha) and decrease the expression of H^+^/K^+^-ATPase [55] (Figure 1, pathway 9).
4. Suppression of acid secretion by neural inhibition of enterochromaffin cell histamine secretion and antral G-cell gastrin secretion [56].
5. *H. pylori* VacA disrupts the incorporation of tubulovesicles (which contain H^+^/K^+^-ATPase) into the gastric parietal cell apical membrane [54].

Acute hypochlorhydria or “epidemic hypochlorhydria” in children was first described by Sir William Osler in 1910. This occurs during initial *H. pylori* infection but is not due to loss of parietal cells. Parietal cell loss only occurs later in established *H. pylori* induced chronic atrophic gastritis, with associated compensatory G-cell hyperplasia and hypergastrinaemia. The mechanisms behind parietal cell apoptosis in *H. pylori* related chronic atrophic (corpus) gastritis include Th1 cytokines (TNF-α, IL-1β), Th17 immune responses involving IL-17A cytokine-ligand induced caspase activation, and anti-parietal cell antibody (APA) generation [57].

### 3.5. H. pylori and Chemotaxis

Chemotaxis is essential for *H. pylori* organisms to find a niche and survive in the hostile environment of the stomach. Chemotaxis for acidity, bicarbonate, urea, energy, amino acids (arginine, glutamine, histidine) and transition metals (iron, zinc, copper, nickel) occurs via *H. pylori* chemosensing and flagellar motor switches. Thus *H. pylori* are able to sense these agents in the gastric microenvironment and swim towards nutrients and away from acidic areas. There are four known *H. pylori* chemoreceptors or transducer like proteins (Tlp), three of which are membrane localized (TlpA, TlpB, TlpC), and one (TlpD), which is wholly cytoplasmic [50,51,52,53,54,55,56].

1. TlpA is able to sense gastric mucosal arginine/amino acids, acidic pH and bicarbonate
2. TlpB senses urea and bacterial quorum (via auto-inducer (AI-2))
3. TlpC senses lactate
4. TlpD senses ROS, alkaline pH, neutrophil derived HOCl and inhibitors of electron transport (Figure 1, pathway 2).

Chemoreceptors and flagellar rotation enable *H. pylori* to rapidly and preferentially colonize the antral and body mucosal glands within 14 h of ingestion, but also areas of injured gastric epithelium and neutrophil activity where nutrients may be more available. *H. pylori* appear to target specific gastric epithelial cell types via chemotaxis, including LGR5+ adult stem cells located in antral glands. This preference for colonization of gastric glands under the gastric mucus is related to the higher pH in the glands, due to buffering by epithelial bicarbonate excretion. By transducing the level of gastric acidity, and being repulsed from areas of low pH (gastric lumen) and attracted toward bicarbonate (gastric glands), *H. pylori* are able to survive, whereas other enteric bacterial pathogens such as *Salmonella* and *Vibrio* spp. cannot [58,59]. After ingestion and colonization of an acid secreting stomach, the majority of *H. pylori* can be found swimming in the first 15μm of the antral mucus, with 30% of *H. pylori* bacteria in the 1–5 μm of the mucus adjacent to the antral epithelium. Only about 2% of *H. pylori* are adherent to the epithelial cells. There are negligible numbers found in the middle or luminal sections of the gastric mucus. In conditions of chronic achlorhydria induced by AG or PPI use, *H. pylori* bacteria are able to colonize the stomach more widely, including the gastric body and fundus [60,61,62,63,64] (Figure 1).

### 3.6. Non-Enzymatic Effects of H. pylori Urease

*H. pylori* urease is a virulence factor with separate and independent effects to its enzymatic activity. Extracellular UreB is able to bind to epithelial cell receptor CD74 (MHCII), which activates pro-survival genes and inflammatory pathways. These include increased:

1. release of IL-8, a chemokine for inflammatory cells including monocytes and neutrophils
2. activation of NF-κB pro-inflammatory pathways
3. activation of primary mucosal macrophages
4. release of gastric epithelial cell Th1 cytokines (IL-1β, IL-6, TNF-α, IL-8)
5. disruption of gastric epithelial tight junctions
6. release of IL-4 and antibody production by splenic lymphocytes
7. platelet aggregation [53].

UreB also binds to TLR-2 receptors on the gastric epithelial apical membrane which is independent of *H. pylori* lipopolysaccharide (LPS) binding. This activates both NF-κB and PI3K/AKT/mTOR/HIF-1α pathways. The induction of gastric epithelial HIF-1α under normoxic conditions by UreB has effects on cell cycling via Cyclin D1 and immune tolerance via Treg cells, and does not appear to involve the usual canonical transcriptional targets of HIF-1α (LDH, VEGF, GLUT1) [48] (Figure 1, pathway 10).

### 3.7. H. pylori Induction of HIF

Reactive oxygen species accumulation and vitamin C deficiency related to *Helicobacter* gastritis are further mechanisms for the normoxic stabilization of HIF-1α, as ascorbate is a required co-enzyme for the activity of the HIF-α prolyl hydroxylase family (PHD). Specific HIF-α proline residues are hydroxylated by HIF-α-PHD, which increases the affinity of the HIF-1α peptide for the von Hippel–Lindau protein (pVHL)-elonginB-elonginC (VBC) complex. HIF-1α is ubiquinated by the VBC complex and then degraded by the 26S proteasome. During normal physiological conditions, there is minimal cellular HIF-1α. However, under hypoxia, ROS or succinate accumulation, HSP activation, mitochondrial dysfunction, vitamin C or iron deficiency, PHD2 is inhibited. HIF-1α is thus stabilized and accumulates within the cytoplasm. HIF-1α is then translocated to the nucleus where it binds with HIF-β to form the HIF heterodimer. HIF activates the transcription targets of the hypoxia response element (HRE), including proliferation (Caveolin-1, CTGF, IGFBP3, MET), angiogenesis (EPO, PDGFβ, VEGF), redox homeostasis (GPX3, HMOX1, SOD2), glucose transport and metabolism (GLUT, HKII, LDHA, PDK1, PGK1, PKM2), epithelial mesenchymal transition (SNAIL, SLUG, VM, ZEB), and metastasis and invasion (CXCL12, CXCR4, LOX, MMP1, MUC-1, S100A4, TWIST1) [38,65].

### 3.8. H. pylori and Correa Pathway

**Figure 5:**
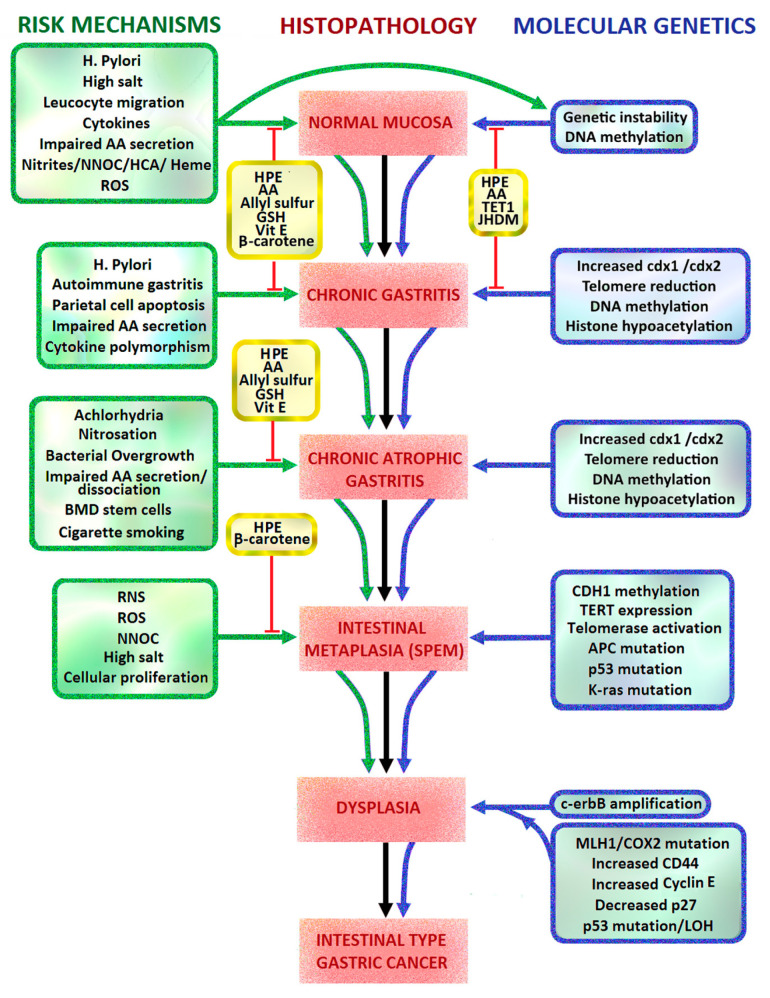
Modern Correa pathway of intestinal-type gastric carcinogenesis, with risk factors and host mechanisms in green, molecular genetics in blue, and preventive factors in yellow. As histological changes progress, antioxidant systems and DNA repair mechanisms become less effective and the epigenetic effects of *Helicobacter pylori* infection and environmental carcinogens more difficult to reverse. AA: ascorbic acid; BMD: bone marrow derived; cdx: caudal type homeobox gene; c-erbB: human epidermal growth factor oncogene; CDH1: E-cadherin gene; GSH: glutathione; HPE: *Helicobacter pylori* eradication, LOH: loss of heterozygosity; SPEM: spasmolytic polypeptide-expressing metaplasia; TET1: ten eleven translocation methylcytosine dioxygenase 1 [6,19,26].

In 1892, the Italian pathologist Guilio Bizzozero reported his observation of spiral shaped bacteria present in the gastric mucosa of dogs. His findings were largely ignored due to the pervasive belief that bacteria could not survive in the acidic milieu of the stomach [66]. In 1975, Paleyo Correa originally proposed the histological pathway for intestinal-type gastric cancer. After the rediscovery of *H. pylori* by Marshall and Warren in 1984 and further research by Correa and others, he incorporated positive risk factors (*H. pylori* infection, dietary salt, N-nitroso compounds) and negative risk factors (vitamin C, β-carotene) for gastric cancer into this pathway in 1992 [6] (Figure 5). The *H. pylori* bacterium was then classified by the WHO in 1994 as a class 1 carcinogen for gastric cancer [7]. Much of the evidence for the role of *H. pylori* in gastric carcinogenesis comes from epidemiological studies in endemic areas.

For example, Uemura et al. conducted a prospective study of 1526 Japanese patients who had duodenal ulcers, gastric ulcers, gastric hyperplasia, or non-ulcer dyspepsia at the time of enrolment [67]. After a mean of 7.8 years of follow-up, 2.9% (36/1246) of those with *H. pylori* and 0% (0/280) of *H. pylori* negative patients developed gastric cancer. Gastric cancers developed in:

1. 21 (4.7%, *p* < 0.001) of the 445 patients with non-ulcer dyspepsia,
2. 10 (3.4%, *p* = 0.002) of the 297 with gastric ulcers,
3. 5 (2.2%, *p* = 0.02) of the 229 with gastric hyperplastic polyps, and
4. 0 of the 275 with duodenal ulcers.

Abnormalities at initial endoscopy associated with future development of gastric cancer included severe grade of gastric atrophy (RR = 4.9), pangastritis (RR = 15.6), corpus predominant gastritis (RR = 34.5) and intestinal metaplasia (RR = 6.4) [67]. Duodenal ulcer patients have corpus sparing, antral specific *Helicobacter* gastritis with high gastric acid secretion, whereas gastric ulcer and gastric cancer patients have fasting hypochlorhydria, low pepsinogen I levels (≤70 ng/mL) and pepsinogen I/II ratios (≤3), and severe, corpus predominant atrophic gastritis [68,69]. Eslick et al. found *H. pylori* conferred a 2-fold risk of developing gastric cancer [70], which correlated with the results from the European Prospective Investigation into Cancer and Nutrition (EPIC)-EURGAST study [71].

### 3.9. H. pylori-Induced Inflammatory Response

*H. pylori* causes gastric mucosal damage by its production of urease, protease, phospholipase, peptidoglycans, ammonia, Hp-NAP, VacA, CagA, BabA, SabA, GGT and acetaldehyde. It also generates reactive oxygen species, reactive nitrogen species and nitric oxide synthase (NOS) by initiating a host inflammatory reaction [72]. This includes a severe host inflammatory response triggered by tumour necrosis factor apoptosis inducing ligand (TRAIL), phosphorylation of IL-1 receptor-associated kinases (IRAK-1) and NF-κB activation [73]. The magnitude of the inflammatory response to *H. pylori* infection is increased by polymorphisms in host cytokine expression, with IL1RN, IL1β-511 and TNFA-308 genotypes being more highly associated with achlorhydria, chronic atrophic gastritis, intestinal metaplasia and gastric cancer [74] (Figure 5).

### 3.10. H. pylori and ROS

**Figure 6:**
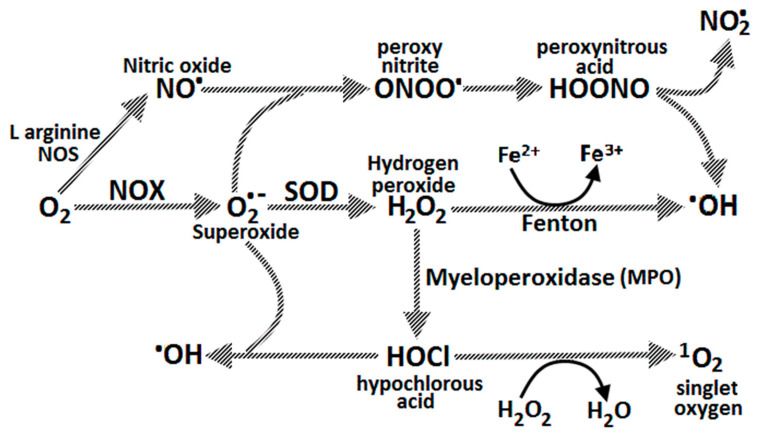
The three sources of DNA damaging hydroxyl radicals; from nitric oxide (NO) reacting with superoxide radicals to form peroxynitrite, from Fenton reactions with hydrogen peroxide (H_2_O_2_) and ferrous iron, or from superoxide reacting with hypochlorous acid (HOCl) from neutrophils. Nitric oxide synthase (NOS), Superoxide dismutase (SOD), NADPH oxidase (NOX), hydroxyl radical (OH**·**). Adapted from Knaapen et al. with permission [76].

Once a virulent strain of *H. pylori* is established in the stomach, *Helicobacter pylori* neutrophil activating protein (Hp-NAP) and chemokine release (IL-8) promote neutrophil, macrophage and T lymphocyte migration into the gastric mucosa. This induces a cascade of further inflammation, respiratory bursts of ROS formation, mucosal injury, DNA damage, parietal cell apoptosis and potential gastric carcinogenesis. DNA can be damaged by depurination, deamination, methylation or oxidation by ROS [33]. The induction of ROS release from gastric mucosal neutrophils by *H. pylori* is 10 times greater than that of *S. aureus* or *E. coli* [75]. The four oxidative agents produced by neutrophils are NO^•^, O_2_^−•^, H_2_O_2_ and HOCl, which interact with each other to form peroxynitrite (ONOO^−^) and hydroxyl radicals (OH**·**). HOCl is an effective killing agent of most human pathogenic organisms, including spore and non-spore forming bacteria. However, the oxidative molecules released by neutrophils and macrophages against *H. pylori* organisms attached to the gastric mucosa cause collateral damage to the epithelial cells (Figure 1 pathway 6, Figure 6).

Initially, O_2_^−•^ is generated by cellular NADPH-oxidase (NOX) in response to phagocytosis of bacterial pathogens, which is then released into the extracellular space and converted to H_2_O_2_ by superoxide dismutase (SOD). H_2_O_2_ is easily diffusible across membranes, and can form OH**·** by Fenton reactions. Neutrophil myeloperoxidases use much of the generated H_2_O_2_ to catalyse the reaction of Cl^−^ + H_2_O_2_→HOCl + OH^−^. HOCl can also react with O_2_^−•^ to form hydroxyl radicals. NO (produced from arginine and oxygen by iNOS) can react with O_2_^−•^ to form peroxynitrite, which subsequently generates OH**·** and NO_2_**·** formation. OH**·** causes much of the DNA base damage in mammalian cells, as well as peroxynitrite. HOCl is known to produce DNA damage such as pyrimidine oxidation products, DNA-protein cross-links and chlorination of DNA bases (e.g., 5-chlorouracil) [76,77] (Figure 1 pathway 12, Figure 6). To evade host inflammatory cell derived ROS damage to *H. pylori* proteins and DNA, *H. pylori* bacteria express DNA and protein repair enzymes and at least 14 antioxidant proteins (e.g., catalase, SOD, thioredoxin reductase) after colonization.

### 3.11. H. pylori and Evasion of Immunosurveillance

The *H. pylori* lipopolysaccharide endotoxin lipid A can be modified to resist host cationic antimicrobial peptides and evade Toll-like receptor 4 (TLR-4) recognition (Figure 1, pathway 5). There are also fucosylated oligosaccharides contained in the O-antigen of *H. pylori* lipopolysaccharide that mimic human Lewis antigens. This assists in *H. pylori* evasion of host T cell immunosurveillance and persistent gastric colonization. The expression of these Lewis type antigens can also be extensively varied by *H. pylori* to permit adaptation to different gastric host environments [78]. Similarities also exist between human heat shock protein 60 (Hsp60) and *H. pylori* Hsp60, leading to generation of anti-Hsp60 autoantibodies. These are associated with diffuse gastric cancers and B-cell lymphocyte mucosa associated lymphoid tissue (MALT) lymphoma [79].

### 3.12. H. pylori and DNA Damage

*H. pylori* causes chronic oxidative stress and MDA formation in the stomach, leading to single and double DNA strand breaks, failure of DNA repair and DNA adducts [80,81]. DNA adducts include 8-hydroxydeoxyguanosine, thymine glycol and 5-hydroxymethyl uracil, which in themselves can cause DNA methylation, common mutations, epigenetic alteration and contribute to gastric carcinogenesis [75].

### 3.13. H. pylori, iNOS, ROS and DNA Hypermethylation

Inducible nitric oxide synthase (iNOS) is the enzyme that synthesizes NO from the amino acid arginine. NOS is inducible in gastric mucosa and neutrophils by *H. pylori* infection, resulting in a significant increase in local NO. Nitric oxide reacts with O_2_^−•^ and metals to form peroxynitrite which, together with epithelial derived H_2_O_2_, can create DNA oxidative adducts. NO also prevents the repair of DNA by 8-oxoguanine glycosylase. Oxidative stress related to *H. pylori* activates oncogenes and inactivates tumour suppressor genes, via hypermethylation of CpG island promoter genes and increased activity of DNA methyltransferase [14]. Ding et al. (2010) outlined the multiple oncogenic pathways induced by *H. pylori.* These include NF-κB, activator protein-1 (AP-1), PI3K, beta-catenin, E-cadherin (*CDH-1*), Runt-related transcription factor 3 (*RUNX3*) and cyclooxygenase 2 (*COX-2*) by modification of chromatin protein and DNA methylation, leading to epithelial proliferation and gastric cancer [82]. Other promoter genes that are affected by *H. pylori* induced methylation include those of:

1. DNA repair [O-6-methylguanine DNA methyltransferase enzyme (MGMT)],
2. DNA mismatch repair [MutL Homologue 1 (*MLH1*)],
3. Cell cycle [Cyclin Dependent Kinase Inhibitor 2A (*CDKN2A*)],
4. Inflammation [Trefoil factor 2 (*TFF-2*)],
5. Transcription factors [Forkhead Box D3 (*FOXD3*), upstream stimulatory factors (*USF1* and *USF2*), *GATA4*],
6. Autophagy (*ATG16L1*),
7. Tumour suppression [Adenomatous polyposis coli (*APC*), Phosphatase And Tensin Homolog (*PTEN*), lysyl oxidase gene (*LOX*)] [83] (Figure 1, pathway 12).

### 3.14. H. pylori, NF-κB, STAT3, TNF-α, Vitamin C and β-carotene

Vitamin C has been shown to inhibit the *H. pylori* mediated activation of NF-κB and STAT3, and promote tumour suppression in AGS cells in vitro by upregulating transmembrane protein with epidermal growth factor (EGF)-like and two follistatin motifs 2 (TMEFF2); and AGS apoptosis by mitochondrial mediated pathways [84]. In vitro studies have shown β-carotene reduces *H. pylori* induced NF-κB activation and ROS levels in AGS cells by its antioxidant activity, thereby inhibiting tumour necrosis factor receptor-associated factor (TRAF) induced gastric epithelial hyperproliferation. The decrease in ROS generation was mediated by β-carotene inhibition of NADPH oxidase. β-carotene was able to block the *H. pylori* induced degradation of IκBα, thereby retaining NF-κB in the cytoplasm, preventing its nuclear translocation and activation of nuclear transcription targets, including TRAF1 and TRAF2 gene expression. Clinical prevention studies have suggested oral intake of β-carotene may decrease the risk of gastric cancer in *H. pylori* endemic areas by as much as 48% [85].

## 4. *Helicobacter* CagA+/VacA+

### 4.1. CagA+/VacA+

*H. pylori* is geographically distributed into seven distinct populations and subpopulations. The East Asian *H. pylori* strain is more pathogenic with differing cytotoxin associated gene A (CagA) and vacuolating toxin A (VacA) expression than European or African strains [86,87]. Carriage of cag-PAI varies from an almost universal presence in the strains hpEastAsia and hpAfrica1, through an intermediate presence (hpEurope) to complete absence (hpAfrica2) [86,87]. VacA and CagA positivity doubles the odds ratio of gastric cancer. This may explain, in part, the *African enigma*: high rates of *H. pylori* infection but low incidence of gastric cancer in Northern and East African populations [86,87]. There are epidemiological differences in *H. pylori* strains, including increased prevalence of host colonization, bacterial virulence and subsequent atrophic gastritis and gastric cancer [86,87].

### 4.2. CagA and EPIYA Carriage

Pathogenic CagA positive *H. pylori* strains are more prevalent in high gastric cancer risk countries such as Japan, South Korea, China (90–95%), Colombia (70%) and lower in North Africa and other low gastric cancer risk areas (USA, Western Europe, Australia (40%)). Induction of gastric carcinogenesis may be related to variations in CagA tyrosine phosphorylation motifs (glutamate-proline isoleucinetyrosine-alanine, (EPIYA)). There are four current CagA EPIYA motifs (A, B, C, D), EPIYA A and B being ubiquitous across the world and EPIYA-C mainly found in *H. pylori* strains from Western countries. Most Western *H. pylori* isolates contain only one EPIYA-C motif (EPIYA-ABC genotype), whilst some have two or three EPIYA-C repeats (EPIYA-ABCC and EPIYA-ABCCC). *H. pylori* strains carrying extra EPIYA-C motifs are more closely associated with gastric cancer. This is in part related to the CagA EPIYA-ABCCC motif inducing increased transcription of *erbB2*, *HGF-R*, *FGFR4* and *TGF-β* growth factor genes and expression of *β-catenin*, *mmp7* and *etv4* genes, and decreased *APC* gene expression in host cells [88]. EPIYA-ABD is found almost exclusively in the virulent East Asian *H. pylori* strain (China, Korea, Japan), which induces higher levels of IL-8 release from gastric epithelium than EPIYA A, B or C expressing strains [86,87]. CagA related IL-8 release increases the host inflammatory response, by attracting inflammatory cells via chemotaxis [89] (Figure 1, pathway 8).

### 4.3. CagA and Type IV Bacterial Secretion System (T4SS)

The oncoprotein CagA is a marker for the *H. pylori* cag-pathogenicity island (cag-PAI), which contains 31 genes including *cagA*, *cagB*, *cagC*, *cagL*, *cagM*, *cagI*, *cagY*, encoding the type IV bacterial secretion system (T4SS). The T4SS is used for the translocation of bacterial products directly into the host epithelial cell cytoplasm, including heptose 1,7-bisphosphate from LPS, chromosomal DNA, peptidoglycan and the cagA gene product. This is achieved by *H. pylori* excretion of the serine protease high temperature requirement A (HtrA), which disrupts epithelial cell-to-cell tight junctions (TJ) by fragmentation of junction proteins occludin and claudin 8, and cleavage of E-cadherin-based cell-to-cell adherens junctions (AJ). Paracellular transmigration of *H. pylori* then enables the T4SS needle-like bacterial pilar binding of the α5β1 and α5β6 integrins in the basolateral cell membrane, and injection of CagA into gastric epithelial cells. The T4SS can also deliver CagA products by interacting with the carcinoembryonic antigen-related cell adhesion molecule family (CEACAM) on host cell membranes via the HopQ adhesin. HtrA is associated with a 40% decrease in E-cadherin levels in *H. pylori* infected epithelial cells, representing a severe disruption to the normal gastric mucosal barrier [90] (Figure 1, pathway 7).

Injected *H. pylori* peptidoglycan is recognized as a pathogen associated molecular pattern (PAMP) by cytosolic nucleotide binding and oligomerization domain 1 (NOD1). NOD1 then associates with the receptor-interacting protein serine-threonine kinase 2 (RICK). This results in activation of NF-κB, translocation of NF-κB to the nucleus, and triggering of a pro-inflammatory response via IL-8 and Chemokine (C-X-C motif) ligand 2 (CXCL2) release (Figure 1, pathway 8). Cellular proliferation is also activated by nuclear NF-κB via human β-defensin-2 (HBD-2) and CXCL2. HBD-2 is a cationic antimicrobial peptide which interacts electrostatically with the negatively charged phospholipid cell membranes of Gram-negative bacteria including *H. pylori*, leading to increased bacterial cell membrane permeability and bacterial death. The antimicrobial activity of HBD-1 and HBD-2 can be completely inhibited by high salt conditions, which may influence *H. pylori* colonization [91] (Figure 1, pathway 11).

### 4.4. CagA and Src

Once *H. pylori* CagA is injected into the gastric epithelial cell cytoplasm, the EPIYA motif is phosphorylated by tyrosine kinases (Abelson murine leukemia viral oncogene homolog 1 (c-Abl) or Src family tyrosine kinase (SFK)). The phosphorylated EPIYA-A or B forms a binary complex with C-terminal Src kinase (Csk), which then phosphorylates and inactivates SFK. Thus, *only* the EPIYA-C or D motifs are able to activate the Src-Homology 2 (SH2) domain inositol phosphatase (SHIP2), which results in downstream Ras/MEK/ERK signaling, NF-κB nuclear translocation, focal adhesion kinase (FAK) inactivation and re-organization of the cellular actin cytoskeleton. This leads to transformation of gastric epithelial cells from a uniform polygonal shape to an elongated state with needle like projections (*hummingbird phenotype*), EMT, expression of mesenchymal markers (SNAIL, vimentin, and ZEB1) and stem cell (CD44) markers, uninhibited proliferation and reduced apoptosis [86,87,92] (Figure 1, pathway 8).

### 4.5. CagA, Vitamin C and Epigenetic Programming

CagA positivity is strongly associated with hypermethylation of genes responsible for cell adhesion and cell cycle control, such as E-cadherin (*CDH-1*), *PTEN*, and *CDKN2A* [93]. Methylation of DNA cytosine, which involves CpG dinucleotides (CIMP), and also histones results in epigenetic silencing of key suppressor genes. Eradication of *H. pylori* has been shown to reduce oxidative stress and DNA methylation levels, including that of *CDH-1* [94]. DNA *demethylation* involves conversion of 5-methylcytosine (5-mCyt) to 5-hydroxymethylcytosine (5-hmCyt), which requires the ten-eleven translocation (TET) family of α-ketoglutarate-dependent dioxygenases (α-KGDDs). CagA positivity is strongly associated with loss of TET methylcytosine dioxygenase 1 (TET1) and decreased expression of PTEN [95]. The TET DNA hydroxylases require 2-ketoglutarate and ferrous iron (Fe^2+^) for their catalytic activity. Ascorbic acid reduces the inactive ferric (Fe^3+^) form of iron to ferrous iron (Fe^2+^) in the α-KGDD complex, in a similar way to the prolyl hydroxylase and lysyl hydroxylase dioxygenase enzymes, required for collagen production. Hydroxylation of 5-mCyt by TET thus requires ascorbic acid as a critical co-factor. Both pharmacological and physiological vitamin C administration has been shown to enhance DNA demethylation in haematological malignancies (AML) and solid cancers such as gastric and colorectal cancer [96,97,98]. Ascorbate is also required for the continued function of Jumonji-C domain-containing histone demethylases (JHDMs), as well as other α-KGDDs which regulate metabolism, DNA repair and DNA/RNA de-methylation. By removing the methyl groups on the methylated lysines in the tail of histone H3, JHDMs can reprogram the epigenetic effects of oxidative stress on chromatin regulation, somatic cells, and gastric carcinogenesis [98,99] (Figure 5).

### 4.6. CagA, E-Cadherin and EMT

*H. pylori* CagA is known to promote carcinogenesis by the induction of EMT, inhibition of apoptosis and the acquisition of stem cell properties by transformed gastric epithelial cells. This is achieved by the effects of chronic inflammation on TGF-β and NF-κB release, and the dysregulation of tumour suppressor pathways (p53, CDH1/E-cadherin, APC, MGMT, MLH1, CDKN2A) and canonical tyrosine kinase signaling pathways. These include receptor associated tyrosine kinases (EGFR/ErbB, HGFR/c-MET) and non-receptor kinases (Abl, JAK, FAK, c-src/Ras/MEK/ERK) [100]. Such pathways are normally involved in the regulation of organogenesis, tissue homeostasis, apoptosis and wound healing, but can be ‘hijacked’ by the CagA oncoprotein, resulting in gastric neoplasia [101,102,103].

E-cadherin is the major transmembrane glycoprotein of the adherens junction, and is bound to the catenins (α-, β-, γ- and p120), APC protein and cytoskeletal actin. E-cadherin maintains intercellular adhesion, tissue architecture, cellular polarity and acts as a tumour suppressor by inhibiting EMT. Unphosphorylated CagA directly interacts with E-cadherin, resulting in disruption of the E-cadherin/catenin/actin cytoskeleton/APC complex, and thence aberrant β-catenin and p120 catenin translocation to the cytoplasm and nucleus. This process is *independent* of Src related tyrosine phosphorylation of CagA (Figure 1, pathway 7). APC is associated with the adherens junction and normally acts as a tumour suppressor by degrading free β-catenin and preventing its nuclear translocation. Nuclear translocation of β-catenin in gastric epithelial cells leads to the transactivation of β-catenin dependent carcinogenesis pathways (cyclin D1, c-MYC, CDX1). Stimulation of *cdx1* gene by nuclear β-catenin induces expression of CDX1, the intestinal differentiation marker MUC1, stem cell proteins CD44, SOX2, Oct4, and Nanog, and EMT markers (vimentin, SLUG). This results in transformation of gastric mucosa into a stem cell like phenotype with intestinal metaplasia, progression to intestinal type gastric cancer, spheroid formation and chemotherapy resistance to 5-FU and cisplatin [104,105].

Nuclear translocation of p120 catenin promotes increased expression of matrix metalloproteinase-7 (MMP7) or “matrilysin”. MMP7 is a zinc dependent endopeptidase which degrades the extracellular matrix and basement membrane by proteolytic cleavage of type IV collagen, casein, gelatins, proteoglycans and fibronectins. This enables epithelial cells to detach and migrate from the basement membrane. MMP7 also directly promotes angiogenesis and progression of gastric cancer. Cytoplasmic p120 catenin interacts with Rho GTPases, which are involved in re-organisation of the cytoplasmic actin cytoskeleton, promoting gastric epithelial cell motility and the ability to metastasize. Release of soluble E-cadherin from the adherens junction can also activate EGFR/ErbB membrane receptors and the Ras/MEK/Erk pathway [106,107,108] (Figure 1, pathway 7).

CagA affects promoter genes important in initiation of gastric carcinogenesis. This includes promotion of EMT via ZEB1 transcription and disruption of the normal homeostatic control of gastric epithelial stem cell differentiation via the Hippo/LATS2/YAP1/TEAD pathway [109]. Cation transport regulator 1 (CHAC1) overexpression in human gastric epithelial (AGS) cells infected with CagA-positive *H. pylori* has been shown to degrade glutathione via glutamylcyclotransferase activity, leading to accumulation of ROS [110]. CagA also interferes with the tumour suppressor apoptosis-stimulating protein of p53 2 (ASPP2), also known as Bcl2-binding protein (Bbp)/tumor suppressor p53-binding protein 2 (p53 BP2), and CHAC1 causes loss of function somatic mutations in the TP53 tumour suppressor gene [101,102,103,110,111]. CagA can increase the degradation of TP53 by E3 ubiquitin ligases, ARF-BP1 (ARF-binding protein 1) and HDM2 (human double minute 2), leading to failure of apoptosis, resistance to anoikis, EMT and gastric stem cell generation [101].

A fraction of CagA is localized to the AGS mitochondria, where it generates ROS production by mitochondrial electron transport complex I and III. These ROS are not able to be scavenged by such antioxidants as the GSH precursor N-acetyl-cysteine (NAC), catalase, allopurinol, or desferrioxamine. Buildup of ROS results in decreased PHD activity; increased stabilization and transcription of HIF-1α; and degradation of SIRT3. This leads to activation of hypoxia response element (HRE) downstream targets, including expression of *VEGF*, *LDHA* and *PDK1*, even under normoxic conditions, which promotes the initiation and progression of gastric neoplasia [112].

### 4.7. CagA and Inflammation

CagA induces activation and migration of T cells into the gastric mucosa [113] as well as increasing the release of inflammatory mediators such as TNF-α, NADH oxidase (NOX 1), pathogen inducible nitric oxide synthase (iNOS), IL-1β, IL-8 and IL-10 [114]. Increased release of gastric mucosal TNF-α and IL-1β is associated with suppression of parietal cell HCl release and gastric achlorhydria. Because of this enhanced inflammation, immunogenicity and cytotoxicity, CagA positive *H. pylori* strains are associated with a significantly increased risk of chronic atrophic gastritis and intestinal metaplasia (OR, 3.48; 95% CI, 1.02 to 12.18); and progression to gastric cancer (OR, 1.64; 95% CI, 1.21 to 2.24) [6]. *Helicobacter* induced chemotaxis for neutrophils appears to be independent of CagA, and more related to Hp-NAP binding to host epithelial cell membrane TLR-2, which induces activation of a Th1 immune response [115]. Tumour associated neutrophils (TANs) induce EMT in gastric epithelium by the release of IL-17a and activation of IL-17a/JAK2/STAT3 signaling [116] (Figure 1, pathway 6).

### 4.8. VacA

VacA, produced by most *H. pylori*, is a pore forming toxin which increases cell membrane permeability, stimulates cell membrane tyrosine kinase receptors and decreases glutathione levels. Glutathione has several important actions including free radical scavenging properties, detoxifying toxins such as heterocyclic amine (HCA) metabolites and reducing DHA back to its active form ascorbate [117,118]. There are numerous gastric epithelial cell surface receptors for VacA including:

1. Receptor-like protein tyrosine phosphatase α (RPTPα),
2. Receptor-like protein tyrosine phosphatase β (RPTPβ),
3. Epidermal Growth Factor Receptor (EGFR),
4. Lipid raft/glycosylphosphatidylinositol-anchored proteins (GPI-AP),
5. Sphingomyelin (SM),
6. Fibronectin (FN),
7. Heparin (H) and heparan sulfate (HS),
8. Low-density Lipoprotein Receptor-related Protein-1 (LRP1),
9. and one T lymphocyte receptor: CD18 [119].

### 4.9. VacA and Vacuolation

VacA induces the formation of cytoplasmic vacuoles in gastric mucosal cells, which makes plasma membranes more permeable and thus susceptible to injury. VacA can be internalized into the gastric epithelial cell by pinocytic-dependent and clathrin-independent endocytosis and cause mitochondrial dysfunction by reducing the transmembrane potential and releasing cytochrome C. This leads to oxidative stress and mitochondrial mediated apoptosis in gastric epithelial cells. After internalization of VacA, vacuolation of gastric epithelial cells occurs due to the accumulation of ammonium ions and water influx via osmosis into the gastric cell endosome. Thus, the pathogenicity of VacA is dependent on the production of ammonia by *H. pylori* urease and GGT [58] (Figure 1, pathway 4).

### 4.10. VacA and CagA

VacA contributes to an immunotolerant gastric microenvironment by blocking T-lymphocyte proliferation and reducing IL-2 production [119]. VacA also activates protein kinases including p38/MAPK, ERK1/2 and downstream activation of VEGF. VacA is not part of the cag-PAI but may act synergistically with CagA to enable persistent colonization and iron extraction from gastric mucosal cells, as well as the activation of AP-1. AP-1 regulates the expression and recruitment of cytokines such as IL-8, IL-6, TNF-α and NF-κB. Variations in *vacA* expression also influence *H. pylori* virulence-the *s1* or *m1* genotypes being more significantly associated with AG and IM [6,119,120]. Most CagA positive *H. pylori* strains carry the toxigenic s1 VacA form and OipA adhesin, while CagA negative strains carry the non-toxigenic *s2 vacA* form without OipA. There is also some evidence that VacA and CagA can have reciprocal antagonism, with CagA inhibiting VacA induced vacuolation and apoptosis, and VacA inhibiting the CagA induced hummingbird proliferative phenotype in gastric epithelial cells [119,121].

## 5. *Helicobacter* Adhesins, Blood Group and Vitamin C

The *Helicobacter pylori* outer membrane proteins (OMP) comprise five gene family members. The first and largest family member includes the *H. pylori* outer membrane porin (HOP) adhesin molecules, which adhere to host cell membrane receptors with differing affinity. There are 21 HOP adhesins, including adherence associated lipoprotein A (AlpA), outer inflammatory protein A (OipA), HopQ, HopZ, blood group antigen binding adhesin (BabA) and sialic acid-binding adhesin (SabA) [86]. BabA is encoded by the *BabA2* gene, of which there are numerous variations. BabA binds to H, Lewis b (Leb) and fucosylated ABO blood group antigens, which are expressed not only on erythrocytes but also gastric epithelium. Sialyl-Lewis x and sialyl-Lewis antigens expressed on gastric epithelium are recognized by SabA [122]. Adherence of *H. pylori* via BabA to the gastric mucosa aids in more effective delivery of VacA and CagA by *H. pylori* to gastric epithelial cells via the T4SS. Apical membrane adherence also prevents shedding of *H. pylori* into the gastric lumen and provides a stable nutrient supply to the bacteria from the damaged host epithelium. Blood group O patients with H antigens, and subjects with Leb antigen have a higher incidence of peptic ulcer disease, suggesting enhanced adherence of *H. pylori* to H and Leb antigen on the gastric epithelium. Su et al. found the combination of OipA, BabA, and SabA antibodies gave a 77.3% positive predictive rate in the diagnosis of gastric cancer, suggesting the carcinogenicity of *H. pylori* strains was closely related to expression of adhesin proteins [123] (Figure 1, pathway 3).

Lewis antigens expressed on the surface of red blood cells, endothelium, kidneys, genitourinary and gastrointestinal tract include Le (a+, b−), Le (a−, b+), Le (a−, b−). As well as better binding affinity of BabA to Leb, Lewis antigens are closely interlinked with secretion of ABH antigens. Le (a−, b+) and Le (a−, b−) genotype is associated with ABH secretion, whereas Le (a+, b−) is a non-secretor (i.e., Lewis a patients do not secrete ABH antigens via mucous glands of the gastrointestinal tract). This means that blood group O patients with H antigens but also with Lewis a do not have H antigens on gastric mucosa, and *H. pylori* BabA cannot effectively adhere. Whereas patients with blood group O have excessive bleeding and duodenal ulcer disease due to improved binding with *H. pylori* adhesins, blood group A patients are at greater risk of gastric cancer [124,125,126].

Blood group O patients have greater pro-inflammatory cytokine IL-6 and TNF-alpha response to *H. pylori*, but not anti-inflammatory cytokine IL-10 [127]. A study of 703 patients with gastric cancer and 1465 non-cancer patients by Nakao et al. [128] demonstrated a statistically significant increased risk of gastric cancer in patients with blood group A and a risk reduction associated with blood group B and O patients. Blood group A patients are also more susceptible to pernicious anaemia and chronic atrophic gastritis, and thus more prone to gastric cancer. Furthermore, immune response to tumours in blood group A patients are lower than blood group O patients. This is related to the similarities between blood group A carbohydrate antigens and gastric cancer A-like Thompsen–Friedenreich (TF) antigen, with a resulting lowered host TF agglutinin response [127,129]. Jaff et al. showed in Korean patients that blood group B were least susceptible to *H. pylori* infections, and blood group O patients most susceptible to *H. pylori* induced ulcers [130]. Even before the association between *H. pylori* and chronic atrophic gastritis was discovered, it was known that blood group O patients had greater production of free HCl in the stomach (86%) and higher levels of serum pepsinogen (564 U/mL) as compared respectively to blood group A patients (73%) and 494 U/mL [131]. Wang et al. (2012) showed individuals with type A blood are more prone to being infected by *H. pylori* than blood group B [117]. By means of a wide range of imaging techniques, the study demonstrated enhanced *H. pylori* attachment to erythrocytes with type A antigen.

Patients with blood group A are at increased risk of atrophic gastritis which impairs gastric secretion of vitamin C levels, compared to patients with blood group O without atrophy who tend to have normal luminal vitamin C levels and gastric pH. Diffuse antral gastritis and duodenal ulceration are associated with normal intragastric vitamin C levels compared to patients with pangastritis, body atrophic gastritis or intestinal metaplasia who have low gastric juice vitamin C and achlorhydria. This may explain the observation that patients with *H. pylori* related duodenal ulceration rarely develop gastric cancer [70,114]. Gastric ascorbic acid secretion in patients with severe gastric atrophy (0.56 mL/min, 0.27–1.20) was markedly lower than those in patients with no atrophy (1.51 mL/min, 0.59–3.30) or with mild (1.43 mL/min, 0.53–3.78) and moderate (1.31 mL/min, 0.47–3.16) atrophy (*p* < 0.005). There was a significant negative correlation between ascorbic acid secretion and severity of atrophy (correlation coefficient = −0.43, *p* < 0.005) [42].

Tight adherence to erythrocyte blood group or gastric mucosal carbohydrate antigens also allows iron to be extracted from the host to be utilized by *H. pylori* [117]. This may explain the phenomenon of refractory iron deficiency anaemia in chronic *H. pylori* gastritis, which is most marked in blood group A patients and least in blood group O patients. Iron can also be utilized by *H. pylori* to cause the Fenton reaction—ferrous iron catalyzes hydrogen peroxide and superoxide to form hydroxyl radicals and cause lipid peroxidation and DNA damage:

Fenton reaction: *2Fe^2+^+ 2H_2_O_2_→2Fe^3+^+ 2OH***·**(hydroxyl radical) + *2OH^−^* [112].

Akatsuka et al. showed in rats that the Fenton chemical reaction generates hydroxyl radicals, which causes significant genomic alterations (particularly deletions), contributing to carcinogenesis [132] (Figure 6).

## 6. Vitamin C and *H. pylori*

### 6.1. Chronic Atrophic Gastritis, H. pylori and Vitamin C

*H. pylori* can cause non-atrophic gastritis or chronic atrophic gastritis. Corpus-predominant chronic atrophic gastritis is associated with loss of parietal cells, hypergastrinaemia, anti-parietal cell antibody (anti- H^+^/K^+^ ATPase Ab, anti-intrinsic factor Ab) generation, increased proliferation by gastric stem and progenitor cells, and replacement of normal gastric epithelium by complete or incomplete IM. This is also referred to as “antralization” or “pseudopyloric metaplasia” of the corpus or fundus. Hypergastrinaemia leads to increased expression of COX-2, and of anti-apoptotic proteins, such as Bcl-2 and survivin. This can promote carcinogenesis and angiogenesis in areas of gastric mucosal atrophy and incomplete metaplasia [133] (Figure 1). It is proposed that some of the metaplastic cells arise from zymogenic chief cells recruited back into the cell cycle, which is involved in spasmolytic polypeptide-expressing metaplasia (SPEM). Spasmolytic peptide is a trefoil peptide which is characteristically expressed in normal intestinal mucosa [57,134,135]. Loss of chief cells in AG and IM is reflected by low serum pepsinogen I levels and pepsinogen I/II ratios [114]. Later in the course of the disease, some of the metaplastic cells are derived from bone marrow derived stem cells which have migrated into the gastric mucosa [118] (Figure 5).

Cag-A positive *H. pylori* strains are strongly associated with chronic atrophic gastritis and IM, and are more likely to cause gastric cancer [6]. This may be in part due to chronic achlorhydria, reduced active secretion of vitamin C, unopposed free radical damage in chronic atrophic gastritis, and persistent hypermethylation of promoter genes [114]. Vitamin C is a bi-acid, with two different pKa of dissociation, 4.17 and 11.6. This means that in the acidic environment of the normal stomach (pH < 4), a greater proportion of vitamin C is dissociated to its anion, ascorbate, which is a more powerful antioxidant than its oxidised form DHA [6,136]. In the more alkaline environment of gastric achlorhydria (pH > 4), there is less dissociation to ascorbate and therefore less effective antioxidant protection (Figure 4).

**Figure 7:**
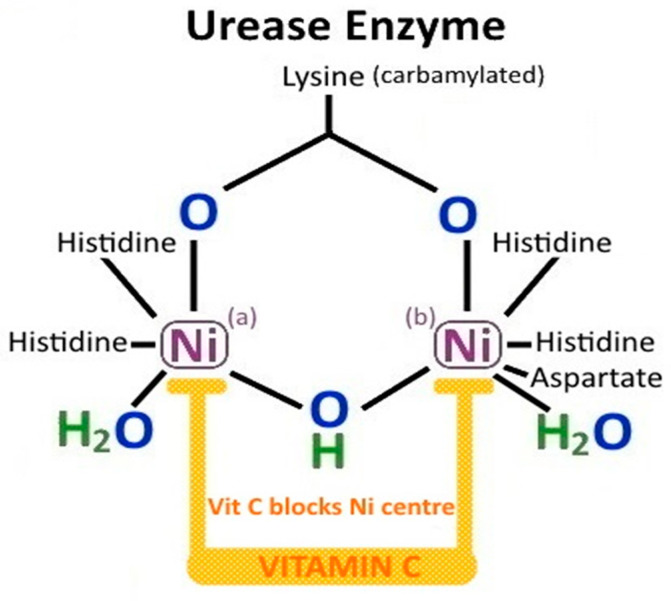
Inhibition of *H. pylori* urease by vitamin C [137].

Vitamin C is a relatively strong acid and contributes to the overall acidity of the human stomach (fasting pH = 2). This is important in host gastric cytoprotection from pathogenic enteric organisms. Vitamin C also helps to protect the gastric epithelium from colonization by *H. pylori* or physical injury by increasing the synthesis of prostaglandin E2 (PGE2) by 90–100%, which stimulates mucus secretion into the gastric lumen. Apart from thiol oxidation of the sulfylhydryl rich urease enzyme by DHA-metal ion complexes, ascorbic acid also reacts with Ni^2+^ found at the centre of *H. pylori* urease enzyme. Ascorbic acid is oxidised to DHA-Ni^2+^ complexes which reduces the dimeric nickel centre and irreversibly inactivates the urease enzyme (Figure 7). This results in the urease molecule becoming unstable and the *H. pylori* organism being unable to create an alkaline environment in which to survive. *H. pylori* is not an acidophile, and if its urease enzymes are inactivated, it can only survive at a gastric pH of 4.0–8.0. Vitamin C has also been shown to improve the eradication rate of *H. pylori* in combination with quadruple therapy (bismuth, metronidazole, amoxicillin, omeprazole), in comparison to standard quadruple therapy (78% vs. 48.8%, *p* < 0.0001). Thus, vitamin C may protect the stomach against initial colonization by *H. pylori* organisms, but may also be useful in eradication therapy for established *H. pylori* gastritis [137].

### 6.2. H. pylori and Gastric Ascorbic Acid Secretion

*H. pylori* associated chronic atrophic gastritis substantially lowers gastric juice ascorbic acid secretion [138]. Waring et al. showed that, whilst plasma and gastric mucosal concentrations of ascorbic acid were unaffected by the presence of chronic gastritis, the gastric *luminal* concentration of ascorbic acid was significantly lower [139]. This was particularly so in vitamin C unsupplemented patients with hypochlorhydria (gastric pH > 4). These patients had a very low median ascorbic acid gastric juice level (9 µmol/L, range 0–82 µmol/L, *p* < 0.05), as compared to unsupplemented patients without hypochlorhydria (gastric pH < 4), who had a much higher median level (39 µmol/L, range 0–483 µmol/L). Oral vitamin C supplementation was less effective in improving luminal gastric ascorbate levels in patients with chronic gastritis than those with normal gastric mucosa [139]. Patients with *H. pylori* infection have a gastric juice ascorbic acid concentration significantly lower than those uninfected (19.3 µmol/L (interquartile range (IQR) 10.7–44.5) versus 66.9 µmol/L (IQR 24.4–94.2), *p* = 0.003). *H. pylori* CagA positive patients have lower gastric juice ascorbic acid concentrations than CagA negative *H. pylori* infected patients (14.8 µmol/L (IQR 7.9–52.2) versus 39 µmol/L (IQR 19.9–142.2), *p* = 0.05) [114].

### 6.3. H. pylori Eradication and Vitamin C

*H. pylori* contributes to diminished gastric luminal ascorbic acid due to consumption by *Helicobacter* phospholipases, proteases and cytochrome c oxidases; increased release of ROS by migrating inflammatory cells; increased gastric pH and impaired active secretion of ascorbic acid due to damaged gastric epithelium. Eradication of *H. pylori* has been shown to improve gastric juice levels of ascorbic acid [140,141]. Sobala et al. [142] showed a significant recovery in intragastric ascorbic acid levels in patients who achieved successful *H. pylori* eradication versus those who failed eradication. The rise in gastric juice ascorbic acid levels was higher in *H. pylori* eradicated patients with final plasma vitamin C levels >30 μmol/L compared to those who were deficient, with plasma levels < 30 μmol/L. It was also found that oral vitamin C supplementation alone was unable to increase gastric juice ascorbate levels in the presence of persistent *H. pylori* infection [142]. Annibale et al. (2003) reported *H. pylori* eradication in patients with chronic superficial *H. pylori* gastritis achieved a decrease in intragastric pH (4 to 2) and an increase in gastric juice ascorbic acid levels (9.02 to 14.48 mcg/mL), but there was no change in patients with existing *H. pylori* related gastric atrophy who achieved *H. pylori* eradication [143]. In atrophic gastritis, *H. pylori* infection also results in gastric achlorhydria, favouring an overgrowth of nitrite-forming bacteria and increasing the formation of nitrite and N-nitroso compounds and the oxidation of ascorbate [144].

### 6.4. Phytochemicals, CagA and Prevention of Gastric Cancer

The effect of CagA induced chronic atrophic gastritis and decreased secretion of vitamin C into the gastric lumen may explain, in part, the variable efficacy of dietary phytochemicals from fruit and vegetables in prevention studies of gastric dysplasia and adenocarcinoma. There have been positive [85,145,146,147], negative and equivocal results [148,149,150,151,152,153], some of which relate to study power and design, *H. pylori* virulence, endoscopic assessment, baseline histology, heterogeneity, controlling for other risk factors, assessment of plasma vitamin C status, dietary recall and length of follow-up [146,147,148,149]. For example, a pooled analysis was performed of 810 prospectively collected non-cardia gastric cancer cases and 1160 matched controls from East Asian countries (Korea, Japan, China), with associated *H. pylori* CagA status, demographic, lifestyle, smoking and dietary data. Higher fruit intake was associated with decreased risk of non-cardia gastric cancer (OR = 0.71, 95% CI: 0.52–0.95, *p*-trend = 0.02). High-fruit consumers without evidence of *H. pylori* antibodies had the lowest odds for gastric cancer incidence (OR = 0.12, 95% CI: 0.06–0.25) when compared to low-fruit consumers who were infected with CagA-positive *H. pylori.* However, the inverse association with high-fruit consumption was lost in individuals infected with CagA positive *H. pylori* (OR = 0.82, 95% CI: 0.66–1.03). The overall low percentage of CagA negative subjects, and the non-smoking status of higher fruit consumers were potential confounding factors in the study [154].

## 7. Probiotics and *Helicobacter* Eradication

The emergence of antibiotic resistance in *H. pylori* bacteria has stimulated interest in the use of probiotics to enhance Helicobacter eradication rates. Live organisms such as *L. acidophilus* and *L. bulgaricus* have been shown to enhance the gastric mucus protection barrier, inhibit *H. pylori* adhesion to gastric epithelial cells, stimulate host immunity, compete with *H. pylori* for nutrients and produce bactericidal agents (SCFA and bacteriocins). Probiotics may increase *H. pylori* eradication rates when used in conjunction with triple therapy, and prevent the loss of microbial diversity in the gastrointestinal tract and thus the side effects of antibiotic treatment. Lactobacillus spp. may be able to decrease oxidative stress in the stomach and the initiation of gastric neoplasia by their production of antioxidants which scavenge superoxides and hydroxyl radicals, and also decrease gastric inflammation by inhibition of *H. pylori* mediated activation of NF-κB, STAT3 and cytokine release (TNF-α, IL-8, iNOS, COX-2). There is some evidence that probiotic therapy may inhibit gastric carcinogenesis by decreasing polyamine synthesis, as polyamines are required for rapid cellular proliferation [155,156,157,158,159,160].

## 8. Dietary and Environmental Risk Factors for Gastric Cancer

**Figure 8:**
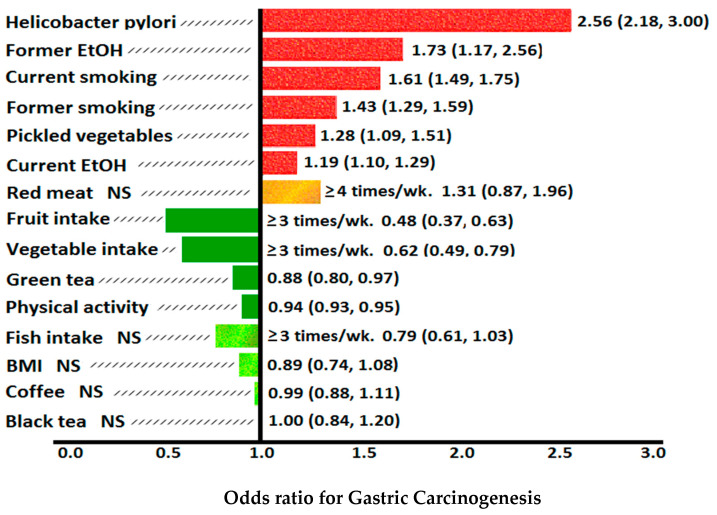
Odds ratios for risk factors and protective factors in gastric carcinogenesis (associations with 95% confidence intervals). Protective factors are shown in green (dark green, significant; light green, non-significant (NS)) and risk factors are shown in red (red, significant; orange, non significant). BMI: body mass index, EtOH: alcohol. Adapted from Poorolajal et al. [164].

Epidemiological studies have shown a diet of pickled, fermented, processed or smoked foods high in nitrites and nitrosamines [161], cooking methods involving high heat such as grilling, broiling and deep frying resulting in heterocyclic amine release [118,162], or diets high in salt, heme iron, red meat [163], or saturated fats can contribute to gastric cancer [164]. Tobacco smoking and alcohol consumption are also risk factors for gastric neoplasia, and may synergistically interact with dietary risk factors and *H. pylori* infection. Increased fruit and vegetable intake is preferable over synthetic vitamin C supplementation as it is likely that ascorbic acid, carotenoids, polyphenols, flavonoids and other phytochemicals act synergistically to prevent gastric neoplasia [36,147] (Figure 8).

### 8.1. Dietary Nitrosamines

Nitrosamines are formed by the reaction of nitrites with secondary amines. Nitrosamines and other N-nitroso compounds (NOCs) are involved in gastric carcinogenesis, and can be derived from exogenous sources (e.g., during cooking, preservation, fermentation or smoking of foods; cigarette smoking), or created endogenously in the stomach. Endogenous synthesis of nitrosamines (ENOCs) accounts for the majority (45–75%) of overall exposure. Consumption of a diet high in pre-formed nitrosamines such as nitrosodimethylamine (NDMA) was shown to increase the risk of gastric cancer (in Swedish women) [165]. However, exogenous NDMA was not shown to be a risk factor (HR, 1.00; 95% CI, 0.7–1.43) in the EPIC-EURGAST study. There are over 300 NOCs, and 90% of nitrosamines are carcinogenic [166]. Stillwell and Correa (1991) measured urinary excretion of nitrate and N-nitrosoproline in 160 subjects in a Colombian population and performed gastric biopsies in 118 of these individuals. The study showed a highly significant association between urinary nitrate, N-nitrosoproline, and intestinal metaplasia and dysplasia, supporting evidence for the role of nitrosamines and other N-NOCs in gastric carcinogenesis [167].

Nitroso compounds can be categorized as C-nitroso compounds (e.g., nitrosoalkanes; R-N=O), S-nitroso compounds (nitrosothiols; RS-N=O), N-nitroso compounds (e.g., nitrosamines; R2N- N=O), and O-nitroso compounds (alkyl nitrites; RO-N=O). N-nitroso compounds can be categorised into nitrosamines and nitrosoamides (includes nitrosocarbamate and nitrosoureas).

Apart from dietary preformed N-nitroso compounds such as nitrosamines, dietary nitrites (NO_2_^−^), used as preservatives in cured meats, beer, bacon, ham and some sausages, have also been implicated in gastric carcinogenesis [168]. Nitrites can produce nitrosylation or nitrosation in the stomach. Nitrosylation leads to the formation of non N-nitroso compounds (adding a nitrosyl ion NO^−^ to a metal such as iron or a thiol). Nitrosation adds a nitrosonium ion NO^+^ to an amine -NH2 leading to formation of nitrosamines such as NDMA or nitrosoproline. This occurs in acidic pH environments, particularly in the stomach (pH < 4); or when intragastric ascorbic acid levels are low. *H. pylori* increases NOS and nitric oxide levels, which promotes the formation of endogenous nitrosamines. *H. pylori* stimulates the macrophage system through the l-arginine/nitric oxide (NO) pathway. Chronic *H. pylori* gastritis may thus increase endogenous NO formation. Subsequent oxidation of NO by ROS results in formation of the nitrosating agents N_2_O_3_ (dinitrogen trioxide) and N_2_O_4_, which can produce nitrosamines or cause other types of DNA damage or lipid peroxidation [169]. Nitric oxide can react directly in the stomach with myoglobin and haemoglobin from red or processed meat to form ENOCs. This does not occur with non-meat protein or inorganic iron. The EPIC-EURGAST study found that the association between ENOCs and non-cardia gastric cancer was only significant in *H. pylori* sero-positive patients (HR, 1.82; 95% CI, 1.32–2.51), and not in *H. pylori* sero-negative patients [170].

Dietary nitrates (NO_3_^−^) and nitrites can lead to formation of N-NOCs and nitrosamines. Nitrites are added to cured meats as preservatives, colour fixatives and inhibitors of spoilage bacteria and food borne pathogens (*Clostridium botulinum)*. Nitrates are ubiquitous in foods, particularly leafy green vegetables (spinach, lettuce, beetroot, arugula, kale, celery). Nitrates are not in themselves carcinogenic but may be partially reduced in the oral cavity by bacteria to nitrites [171,172]. Nitrite does not circulate in the bloodstream because it is oxidized by oxyhemoglobin to nitrate. Nitrates can be converted to nitrites immediately following ingestion. Nitrates can also be absorbed in the small intestines, circulated in the bloodstream, concentrated in the salivary glands (by 10 fold over plasma), secreted in saliva and reduced by anaerobic buccal bacteria to nitrites at a later stage. This is the more common pathway, known as the enterosalivary circulation of nitrates [171]. Approximately 25% of ingested nitrates undergo enterosalivary circulation, which occurs in humans but not in mice or rats. An estimated 20% of the salivary nitrates are converted to nitrites, which, when swallowed, deliver large amounts of post-prandial nitrites to the gastric mucosa. Nitrites can react with gastric acid (HCl) to produce nitrous acid (HNO_2_) which actively nitrosate amides to form nitrosamides (HN_2_O_2_). Two molecules of HNO_2_ can form N_2_O_3_ in the stomach, and then nitrosate amines to form gastric nitrosamines (H_2_N_2_O). Ascorbate reduces HNO_2_ to NO, and also reacts more rapidly with N_2_O_3_ than amines, thereby inhibiting nitrosation. The rate of chemical nitrosation in the stomach increases as the gastric ascorbate/nitrite ratio decreases [161].

Alterations in resident gastric anaerobic microbiota also contribute to nitrosation, in association with the gastric achlorhydria induced by *H. pylori*, AG or long-term proton pump inhibitor use [161]. Commensal bacterial fermentation in the stomach can create high levels of nitrite, particularly in AG when the gastric secretion, or the dissociation, of gastric luminal ascorbic acid is too low to offer protection [139]. At a pH of >5 during gastric achlorhydria, bacterial derived nitrate reductase is thus more important than gastric HCl in the formation of nitrites. This leads to endogenously formed NOCs, and may contribute to gastric carcinogenesis [136]. Gastric microbial dysbiosis, associated with AG, IM and epithelial dysplasia and diagnosed by 16S rRNA gene sequencing, can be reversed after successful *H. pylori* eradication treatment. This suggests that *H. pylori* and altered gastric microbiota have synergistic contributions to gastric carcinogenesis [173] (Figure 5).

### 8.2. Vitamin C, Allium Vegetables and Nitrosamines

The allium vegetables (onions, garlic, shallots) contain allyl sulfur compounds. These inhibit the spontaneous and bacterial mediated formation of nitrosamines (eg nitrosoproline) by favouring the formation of nitrosothiols and decreasing the availability of nitrites. They also block the alkylation of DNA by nitrosamines, which reduces the risk of N-NOC gastric carcinogenesis. The 2007 World Cancer Research Fund meta-analysis of allium vegetable ingestion showed dose related significant decreases in the risk of gastric cancer, but this was not confirmed in the EPIC study. Similarly, vitamin C inhibits the endogenous nitrosation of amines by nitrites in the formation of gastric nitrosamines by 87–100% at a normal gastric pH of 2.5, and promotes nitrosylation of red meat derived heme to nitrosyl-heme, a compound which is not carcinogenic [174,175]. The EPIC-EURGAST study showed the risk of non-cardia gastric cancer from ENOCs correlated with plasma levels of vitamin C. In patients with low plasma levels of vitamin C (<40 μmol/L), the associated odds ratio of ENOCs and non-cardia cancer was 3.24 (95% CI, 1.77–5.93), but there was no association in patients with plasma vitamin C > 40 μmol/L (OR, 1.10; 95% CI, 0.63–1.93). This was thought to be possibly related to lower vitamin C levels in *H. pylori* infected patients. Indeed, when the analysis of the risk of ENOCs and low plasma vitamin C was restricted to *H. pylori* positive patients, the non-cardia gastric cancer risk was even higher (OR, 3.52; 95% CI, 1.8–8.9) [170] (Figure 5).

### 8.3. Heterocyclic Amines

Heterocyclic amines are mutagens formed during the cooking of protein rich foods, particularly meat and fish cooked at high temperature. This process involves protein pyrolysis or the Maillard reaction [118]. Ohgaki et al. showed heterocyclic amines in cooked foods were carcinogenic in mice, rats and monkeys [162]. HCAs are associated with gastric cancer, and also liver, lung, blood vessel, colon and mammary gland cancers, lymphomas and leukaemia.

The consumption of fruit and vegetables rich in antioxidants, polyphenols and other phytochemicals can decrease the formation, bioactivation and carcinogenicity of HCAs and nitrosamines. For example, adding garlic or onions to red meat prior to cooking decreased the formation of the HCAs 2-amino-3,8-dimethylimidazo (4,5-f) quinoxaline (MeIQx) by 66.2–88% and 2-amino-1-methyl-6-phenylimidazo(4,5-b)pyridine (PhIP) by 79–94.3%. After ingestion, HCAs are activated by N-oxidation via cytochrome P4501A2, sulfotransferases and N-acetyltransferases (NAT), to reactive electrophilic species with greater overall genotoxicity than parent HCAs. The activity of NAT mRNA expression in humans can be suppressed by the allium vegetables (e.g., onions, garlic) [174].

Poplawski et al. [118] found that DNA damage induced by HCAs was significantly higher in subjects with *H. pylori* than those who were not infected, suggesting that *H. pylori* facilitates the mutagenic effect of HCAs. However, vitamin C can reduce the toxic effects of HCAs on gastric mucosal cells regardless of *H. pylori* status. HCAs can also be conjugated and detoxified by glutathione which, like ascorbic acid, is a strong antioxidant. VacA produced by *H. pylori* is able to deplete levels of cellular GSH, and therefore increase the exposure of the gastric mucosa to the mutagenic effects of HCAs [58,118].

### 8.4. Salt and H. pylori

Salt has been identified as a risk factor for gastric cancer since 1959, related to the consumption of large amounts of cured meat, soy sauce, salted fish, pickled vegetables and other salt-preserved foods (Figure 8). A meta-analysis by D’Elia et al. of 268,718 subjects showed an increased risk of gastric cancer with increased salt intake [176]. “High” and “moderately high” versus “low” salt intake were both respectively associated with increased risk of gastric cancer (RR = 1.68 [95% C.I. 1.17–2.41], *p* = 0.005 and RR = 1.41 [1.03–1.93], *p* = 0.032). This was particularly so in Japanese subjects and those who had a higher consumption of selected salt-rich foods such as salt fermented fish, fermented soy paste or Korean kimchi (pickled cabbage).

Salt damages the mucous membrane of the stomach leading to inflammation and facilitating colonisation by *H. pylori*. Other reviews have suggested that high salt conditions are involved in gastric carcinogenesis by inducing hypergastrinaemia, endogenous mutations, gastric epithelial cell proliferation, altered gastric mucus viscosity, and potentiating the effects of *H. pylori* CagA and nitrosamines (e.g., *N*-methyl-*N*-nitro-*N*-nitrosoguanidine (MNNG)) [26,177]. Indeed, a 3 fold higher variation in gastric cancer mortality in Japanese prefectures (e.g., Akita (high salt) vs. Okinawa (low salt)), despite similar patient genetic makeup and *Helicobacter* strains, has been ascribed to large differences in dietary salt intake [178]. A significant association between dietary salt and gastric cancer was only observed in Japanese subjects who had both *H. pylori* infection *and* atrophic gastritis (HR, 2.87 [1.14–7.24]) [179]. In a 2015 meta-analysis of 76 prospective cohort studies of dietary risk factors for gastric cancer, every increment of 5 g/day intake of salt was found to increase the risk of gastric cancer by 12% [146] (Figure 5).

Recently, it was found that salt regulated multiple genes encoding *H. pylori* outer membrane proteins (HOP family), including adhesins (SabA, HopQ) and proteins involved in iron acquisition (FecA2 and FecA3). Transcript levels of *H. pylori sabA*, *hopA*, and *hopQ* are increased under high-salt conditions, whereas transcript levels of *H. pylori fecA2* and *fecA3* are decreased under high-salt conditions [180].

### 8.5. Tobacco Smoking, Vitamin C and H. pylori

Tobacco smoking is a significant risk factor for gastric cancer due to ingestion of toxic mutagenic chemicals such as tar (quinone-hydroquinone radicals), cyanide, heavy metals (e.g., cadmium, chromium, nickel); aromatic carcinogens, nitrosamines and nicotine, generation of ROS/RNS and lipid peroxidation [34,180]. Nicotine induced free radicals react with biomembranes causing oxidative destruction of polyunsaturated fatty acid (PUFA), producing cytotoxic aldehydes via lipid peroxidation. Thiocyanates generated from tobacco smoke are secreted in saliva and increase chemical nitrosation in the acidic stomach, particularly at the gastric cardia. The EPIC study found a 73–87% increased risk from smoking, including gastric cardia adenocarcinoma (OR = 4.1), the risk increasing with the intensity and duration of cigarettes smoked. Smoking was also a risk factor in non-cardia gastric adenocarcinoma (OR = 1.94) [181].

Whilst the EPIC study showed smoking was a risk factor for both males and females, Zaridze et al. found smoking increased the risk of developing gastric cancer in males, but not in females [181,182]. The study also showed a dose response relationship, with patients with higher pack year histories being at greater risk. In a Japanese study performed by Koizumi et al. [183], risk of gastric cancer persisted for 14 years after cessation of smoking. Valavanidis et al. [184] suggested that 11% of gastric cancers are associated with smoking. Fruit consumption increased total plasma antioxidant levels in non-smokers but not in smokers, indicating that vitamin C protective effects were attenuated by smoking. However, Bohn et al. showed antioxidant levels were improved by consumption of antioxidant rich foods, even in smokers [185]. Some of the mechanisms by which smoking promotes gastric cancer include reduction of the protective effects of prostaglandin (PGE2) production and gastric mucus integrity [186], GSH and intragastric vitamin C levels [187]. Gonzalez et al. [188] showed smokers had a higher incidence of *H. pylori* infection, which may be the result of, or contributes to, lower gastric luminal vitamin C [187]. Wang et al. [189] demonstrated increased ROS in rat gastric mucosa when exposed to cigarette smoke, suggesting that smoking increased free radical production and absorption. Tobacco smoking doubles the gastric epithelial dysplasia risk compared to non-smokers [190], the risk being greatest in the region of the gastric cardia [181].

Tobacco smoking and *H. pylori* infection may be synergistic in gastric carcinogenesis. A recent prospective study of the association of smoking and *H. pylori* status with gastric cancer risk was performed in 1446 non-cardia gastric cancer cases and 1796 controls from China, Japan and Korea. It was found that current smoking and *H. pylori* sero-positivity increased the risk of non-cardia gastric cancer (OR = 1.46; 95% CI, 1.10–1.93) as opposed to no increased gastric cancer risk amongst *H. pylori* sero-negative current smokers (OR = 0.93; 95% CI, 0.65–1.33) [191].

Jarosz et al. [187] measured plasma and gastric juice total vitamin C levels using spectrophotometry in 86 Polish subjects divided into four groups:

1. non-smokers/*H. pylori* negative: mean gastric vit C = 17.1 mcg/mL,
2. non-smokers/*H. pylori* positive: mean gastric vit C = 12.6 mcg/mL,
3. smokers/*H. pylori* negative: mean gastric vit C = 5.8 mcg/mL,
4. smokers/*H. pylori* positive: mean gastric vit C = 3.9 mcg/mL.

The concentration of vitamin C in gastric juice was significantly lower in smokers than in non-smokers (*p* < 0.05) and *H. pylori* infection in smokers was associated with the lowest gastric luminal vitamin C levels [187]. Pasupathi [34] suggested that ingestion of cigarette smoke containing nicotine, nitrosamines, tar and other mutagenic toxins increased oxidative damage and attenuated the effects of antioxidants such as vitamin C, vitamin E, glutathione and β-carotene.

Smokers may have lower vitamin C levels than non-smokers due to:

1. Consumption of ascorbic acid to DHA by ROS and oxidative stress in the stomach and other tissues exposed to cigarette smoke/tar.
2. Vitamin C is used for regeneration of vitamin E, β-carotene or glutathione which have been directly oxidized during scavenging of free radicals and ROS from cigarette smoke.
3. Smokers have lower dietary consumption of vitamin C containing foods.
4. Cigarette smoke induced oxidative damage of proteins and peroxidation of lipids are accompanied by a marked drop in tissue ascorbate levels.
5. Increased *H. pylori* infection in smokers vs. non-smokers.
6. Failure of regeneration of ascorbate from DHA by GSH-dependent reductases.

As such, the protective effect of ascorbic acid supplementation may only be appreciated in smokers after *H. pylori* eradication, or after smoking cessation. Comparatively large amounts of vitamin C are also required to counteract the high levels of free radicals, lipid peroxidation, thiobarbituric acid, NDMA and F2-isoprostane generated from tobacco smoking (Box 1) [34].

### 8.6. Alcohol

Consumption of alcohol is a risk factor for gastric cancer. In a 2017 meta-analysis of 23 prospective cohort studies published from 1987–2016, every increment of 10g of alcohol consumed per day increased the gastric cancer risk by 7% (95% CI 1.02–1.12; *I*^2^ = 28.9%, *p* = 0.002). The authors commented that there was only one study of alcohol consumption in gastric cancer which adjusted the relative risk of alcohol consumption and gastric cancer relative to *H. pylori* status [192].

The EPIC study showed heavy alcohol consumption (>60g/day) increased the risk of non-cardia intestinal type gastric cancer (OR = 1.65). This has been supported by evidence from Chinese and Russian studies. Ma et al. found heavy drinking (7 times a week), and binge drinking (>55g alcohol intake per occasion) produced a 3.48-fold (95% CI, 1.13–10.73) and 3.27-fold (95% CI, 1.01–10.56) higher risk in *H. pylori* negative subjects. In patients who were *H. pylori* IgG sero-positive, there was no significant association found between drinking pattern and gastric cancer risk [193].

Zaridze et al. [182] showed in a case control study consisting of over 800 subjects that hard liquor consumption, particularly vodka, increased the risk of gastric cardia cancer in males (OR= 3.4, CI = 1.2–10.2) and non-cardia cancer in females (OR = 1.5, CI = 1.0–2.3). Vodka consumption increased the respective gastric cancer risk 2.0-fold (95% CI, 1.2–3.1) and 2.3-fold (95% CI, 1.4–3.7) in subjects who were negative or positive for *H. pylori* infection, compared to their non-drinking counterparts. Moy et al. [194] showed in 391 subjects that >50 g/day of alcohol increased the risk of gastric cancer (HR = 1.4). Metabolites of alcohol (acetaldehyde and acetate) cause a direct toxic effect on the gastric mucosa and also increase the absorption of nitrosamines, including those from tobacco smoke [195]. In a prospective population-based study of 69,962 Norwegian subjects from 1984–2002, combined high use of cigarettes (>20/day) and alcohol (>5 occasions/14 days) increased the risk of non-cardia gastric adenocarcinoma almost 5-fold (HR = 4.90, 95% CI = 1.90–12.62). The confounding effect of *H. pylori* was an unknown factor in this study [196].

Polymorphisms in the Aldehyde Dehydrogenase 2 Family Member (*ALDH2)* gene have been demonstrated to increase the risk of gastric cancer in a Korean population of current or ex-alcohol consumers, as compared to never/rare consumers. This was particularly so in ALDH2*1/*2 carriers [5].

## 9. Family History and Genetic Mutations

Genomic instability caused by various environmental and genetic factors is characterized by microsatellite or chromosomal instability. It has been proposed that greater than 4.2 genomic alterations results in a significant risk of gastric cancer [197]. Gonzalez et al. identified mutations in genes including *XPG*, *PLCE1*, *HFE*, *ERCC5*, *EZH2*, *DOC2*, *CYP19A1*, *ALDH2*, and *CDH1* to be linked with gastric cancer [198]. The risk of gastric cancer is higher in subjects with pre-existing familial cancer syndromes such as:

1. Hereditary Diffuse Gastric Cancer (*CDH1* germline mutation)
2. Breast cancer syndrome (BRCA2)
3. Hereditary non-polyposis colorectal cancer (HNPCC, Lynch II) syndrome (MSH2/MLH1/MSH6 mutation),
4. Li Fraumeni syndrome (TP53 mutation)
5. Familial adenomatous polyposis (FAP) syndrome (APC mutation).

In patients with Hereditary Diffuse Gastric Cancer who were negative for the *CDH1* mutation, other germline mutations have been discovered including *BRCA2*, *STK11* (serine/threonine kinase 11; Peutz–Jeghers syndrome), *ATM* (Ataxia–Telangiectasia mutated), *SDHB* (succinate dehydrogenase complex iron sulfur subunit B), *PRSS1* (Serine protease 1; Hereditary pancreatitis syndrome), *MSR1* (macrophage scavenger receptor 1), *CTNNA1* (catenin alpha 1) and *PALB2* (Partner and localizer of BRCA2) [5,12].

### 9.1. Sodium Dependent Ascorbic Acid Transporters

Mutations in sodium dependent ascorbic acid transporters are associated with an increased risk of gastric cancer. Solute carrier family 23 member 1 (*SLC23A1*) and *SLC23A2* are genes that respectively encode SVCT1 and SVCT2. Wright et al. found single nucleotide gene polymorphisms in *SLC23A1* and *SLC23A2* resulted in low serum ascorbic acid levels, and was associated with a 41% increase in gastric cancer [199]. Another study showed *SLC23A2* polymorphism was associated with gastric cancer but *SLC23A1* polymorphism was not [200].

### 9.2. Glutathione and Gastric Cancer

Genetic aberrations in critical glutathione pathways also increase gastric cancer risk. Zhang et al. [201] showed mutations of the gene expressing glutathione S-transferase P1 (GSTP1), a critical enzyme in the formation of glutathione, resulted in a higher risk of gastric cancer. The most common polymorphism of the *GSTP1* gene is A-->G at nucleotide 313, a mutation which results in substitution of an amino acid (Ile105Val). The risk of gastric cancer was higher in patients with the *GSTP1* substitution mutation, showing that low levels of glutathione increased gastric cancer risk. Subjects with *GSTP1* mutation had further additive risks for gastric cancer with smoking (OR = 1.64), alcohol (OR = 1.64) or *H. pylori* infection (OR = 3.7). CpG hypermethylation of *GSTP1* is an epigenetic modification which is commonly found in EBV related gastric cancer (20%), but rare (0.4%) in EBV-negative gastric cancer [202].

Other important mutations associated with glutathione include polymorphisms of *GSTT1* and *GSTM1*. GSTT1 is important for the conjugation of toxins by glutathione to detoxify compounds in the cytosol, mitochondria and microsomes. Conjugation allows the body to escort toxins out of body via urine or bile. It has been demonstrated that null allele polymorphism of *GSTT1* (22q11.2) increases the risk of gastric cancer because this prevents conjugation of toxins by glutathione. GSTM1 synthesizes and recycles glutathione. Polymorphisms of *GSTM1* have also been shown to increase gastric cancer risk. The GST family members, including GSTM1, are required for the detoxification of carcinogens generated from burning cigarettes, and also metabolites of alcohol. Patients with mutations in both *GSTT1* and *GSTM1* had a significantly increased risk of gastric cancer over those with both wild type genotypes (OR = 1.95, 95% CI: 1.42–2.67; I^2^ = 0%) [202,203].

## 10. Gastric MALT Lymphoma and *H. pylori* Eradication

The majority of gastric MALT lymphomas (92%) have been shown to be associated with *H. pylori* infection [204]. The *H. pylori* strains involved in gastric MALT lymphomas are less virulent than those associated with gastric adenocarcinoma, in that they contain the vacA m2 allele and lack the cag pathogenicity island (cagPAI). CagA positive strains are more likely to be associated with diffuse large B-cell lymphoma (DLBCL) [205]. Marginal zone B cell lymphomas develop in the mucosa of the stomach and are often found to harbour the translocation t(11;18)(q21;q21)—this in fact predicts poor response to treatment. Complete remission (CR) of gastric MALT lymphoma with *Helicobacter* eradication alone has been reported in up to 85% of patients who are *H. pylori* positive. Interestingly, patients who are *H. pylori* negative can also respond to antibiotic therapy for gastric MALT lymphoma, and achieve a CR of up to 57%. *Helicobacter* eradication was successful regardless of the MALT stage or the involvement of bone marrow. MALT lymphoma relapse can occur some years following a CR, and, because of the associated risk of development of gastric cancer, endoscopic surveillance is recommended [204,205,206,207,208,209,210,211,212,213].

## 11. Prevention of Gastric Cancer by *H. pylori* Eradication

### 11.1. Helicobacter pylori Eradication and Gastric Cancer

Successful eradication of *H. pylori* results in significant reduction of future incident rates of gastric cancer in both asymptomatic *H. pylori* positive individuals (pooled incidence rate ratio, 0.62; 95% CI: 0.49–0.79) and post-endoscopic resection of early gastric cancers (pooled incidence rate ratio, 0.46; 95% CI: 0.35–0.60) [213]. In the 2016 meta-analysis of 21 RCTs, *H. pylori* eradication decreased the pooled incidence rate of gastric cancer in a non-linear way with increasing baseline incidence of gastric cancer (*p* = 0.018) [214]. A 2017 meta-analysis of seven RCTs in prevention of primary gastric cancer with *H. pylori* eradication showed a similar reduction in overall relative risk of 0.67 (95% CI: 0.48 to 0.95). However, the respective overall risk difference (RD) was 0.00 ([95% CI: −0.01 to 0.00] and number needed to treat was 125.5 [95% CI: 70.0 to 800.9]). It was thought that the discrepancy between the pooled relative risk (RR) and the RD was because data of rare events was used, and the relative risk overestimated the actual effect [215].

A further 2019 meta-analysis of 31 RCTs showed a significant reduction in future gastric cancer after *H. pylori* eradication (OR = 0.46) in Eastern Asia. On subset analysis this effect was most pronounced in Japanese (OR = 0.39) and Korean (OR = 0.47) patients [216]. Other beneficial effects of *H. pylori* eradication beyond gastric adenocarcinoma prevention include decreased peptic ulcer disease, MALT lymphoma, functional dyspepsia, atrophic gastritis, vitamin B12 and iron deficiency, and idiopathic thrombocytopenia purpura. Due to the potential benefits of *H. pylori* eradication in endemic countries, population based testing commenced in Taiwan in 2004 and Japan in 2013 [4].

Extending population based *H. pylori* testing to countries with a low prevalence of gastric cancer is controversial [217]. There are differences in H. pylori strain virulence and dietary and environmental risk factors in developed nations; and modern guidelines recommend patients found to be infected with *H. pylori* should be treated. This may potentially increase the risk of antibiotic resistance, change gastrointestinal microecology or increase costs. A retrospective cohort study of *H. pylori* diagnosed in 371,813 symptomatic USA Veterans was recently reported [218]. Successful eradication reduced the future risk of non-cardia gastric cancer by 76% (SHR, 0.24; 95% CI, 0.15–0.41; *p* < 0.001) in 38,535 patients with proven *H. pylori* infection on urea breath testing, faecal antigen testing or histopathology. Eradication was only attempted in 75% of these patients (28,818/38,535), which unintentionally provided a sub-group of untreated patients with which to compare outcomes. The overall incidence of gastric cancer in untreated patients was relatively low, respectively 0.37%, 0.5%, and 0.65% at 5, 10, and 20 years after diagnosis of *H. pylori* infection. However, the gastric cancer risk was higher in patients of Black (SHR, 2.0) Hispanic (SHR, 1.59) and Asian (SHR, 2.52) race or ethnicity when compared to Caucasian patients. Male sex, older age (SHR, 1.13) and smoking (SHR, 1.39) were additional significant risk factors [218].

One of the limitations of the study was only 8020 of the 28,818 patients (28%) who received *H. pylori* treatment had post treatment testing. Of these, 90.9% (7292/8020) achieved successful eradication. It was thought that this study could inform future decisions about population based *H. pylori* screening in Western countries with low overall gastric cancer incidence, but higher risk in some demographic subgroups [218]. Post treatment testing to confirm *H. pylori* eradication in the present era of *H. pylori* clarithromycin and metronidazole resistance is emphasized. This is so the preventative effect of *Helicobacter* treatment is not diluted in prospective trials [219].

### 11.2. H. pylori Eradication, Intestinal Metaplasia and Gastric Cancer

A more targeted approach in high risk patients before they develop gastric precancerous changes, as compared to population based *H. pylori* eradication may potentially be more efficacious in the prevention of gastric cancer. Early, successful *H. pylori* eradication may lead to regression of atrophic gastritis but not established intestinal metaplasia [74,219,220]. For example, the preventative effect of *H. pylori* eradication in 1676 *H. pylori* positive, first degree relatives of Korean patients with gastric cancer was recently reported in a double blind, placebo controlled trial with a median follow-up of 9.2 years. Gastric cancer occurred in 5/608 (0.8%) of participants in whom *H. pylori* infection was successfully eradicated and in 28/979 (2.9%) of participants who had persistent infection (HR, 0.27; 95% CI, 0.10 to 0.70) [221]. The observation that *H. pylori* eradication may not improve the risk of future gastric cancer development after long-term follow up in patients with established IM is supported by the finding that CIMP is reduced by *H. pylori* eradication in patients with AG and non-IM mucosa, but not in IM. The incidence of CIMP also appears to progress from AG to gastric cancer, in parallel with the histological changes of the Correa pathway [222] (Figure 5). However, the concept of IM being a “point of no return” in the prevention of gastric cancer is still debated, particularly with the recent reporting from the Shandong Intervention trial [214,223].

### 11.3. Shandong Intervention Trial

The Shandong Intervention Trial was a blinded, randomized, placebo controlled gastric cancer prevention trial involving 3365 participants who underwent initial gastroscopy and *H. pylori* serological testing [223]. The study was commenced in 1995 and conducted in rural Linqu County, Shandong province, northern China, which has a high incidence of gastric cancer mortality (55/10^5^ males, 19/10^5^ females). Linqu county also has a high prevalence of *H. pylori* infection and vitamin C and selenium deficiency. The study evaluated the long-term effect of *H. pylori* eradication (omeprazole and amoxicillin for two weeks), and/or oral 250 mg vitamin C, 100 IU vitamin E, 37.5 μg selenium (taken for 7.3 years) and/or garlic supplementation or their placebos (taken for 7.3 years) on gastric cancer development in 2258 *H. pylori* seropositive people in a 2 × 2 × 2 factorial design. This was compared to 1107 *H. pylori* seronegative people who were given vitamin and/or garlic supplementation or their placebos in a 2 × 2 factorial design. Follow-up urea 13C breath testing in 1996 found 382 of the original 2258 seropositive participants (17%) had persistent *H. pylori*, and they received a further two weeks of Helicobacter eradication therapy [223].

In 2019, the study group reported their 22.3 year follow-up results, which found *H. pylori* eradication treatment significantly decreased gastric cancer incidence (OR, 0.48, 95% CI: 0.32–0.71, *p* < 0.001). Gastric cancer incidence also decreased significantly with vitamin supplementation (OR, 0.64, 95% CI: 0.46–0.91, *p* = 0.01) but not garlic supplementation (OR, 0.81, CI: 0.57–1.13, *p* = 0.22). All three interventions showed statistically significant reductions in gastric cancer mortality: *H. pylori* eradication HR = 0.62 (95% CI: 0.39–0.99, *p* = 0.05), vitamin supplementation HR = 0.48 (95% CI: 0.31–0.75, *p* = 0.001), and garlic supplementation HR = 0.66 (95% CI: 0.43 to 1.00, *p* = 0.05). Hazard ratios were adjusted for baseline histology (moderate chronic atrophic gastritis or less severe gastric lesions, severe chronic atrophic gastritis or superficial intestinal metaplasia, deep intestinal metaplasia, or dysplasia), age, sex, history of ever using alcohol, and history of ever smoking. The beneficial effect of *H. pylori* eradication on gastric cancer incidence and mortality was evident even in older patients (55–71 years), and even in those with intestinal metaplasia and dysplasia at baseline endoscopic assessment. The preventative effect of vitamin supplementation on gastric cancer and gastric cancer mortality was more evident in younger patients (<45 years) or those with more favourable baseline histology (normal, superficial gastritis, chronic atrophic gastritis). Combining vitamin supplementation with *H. pylori* eradication in seropositive subjects appeared to be synergistic in preventing gastric cancer incidence OR = 0.30 (95% CI: 0.16–0.55) and gastric cancer mortality HR = 0.31 (95% CI: 0.15–0.66). The joint effect of garlic administration and *H. pylori* eradication was also greater than the individual effects on gastric cancer incidence OR = 0.38 (95% CI: 0.21–0.69), and gastric cancer related mortality HR = 0.44 (95% CI: 0.23–0.84) [223].

The finding that a long carcinogen-free period was necessary for regression of premalignant mucosal changes and prevention of cancer progression is supported by mouse studies of gastric cancer and *Helicobacter felis*, Mongolian gerbil studies of gastric cancer and *Helicobacter pylori*, and also clinical studies of *H. pylori* eradication. These studies show that early *Helicobacter* eradication was more effective in gastric cancer prevention, and that prolonged anti-oxidant protection (vitamin C, vitamin E, β-carotene, selenium) without cessation was required after initial *Helicobacter* eradication [134,223]. The progression of intestinal metaplasia to dysplasia in the stomach appears to be faster in older patients (>40 years) than in younger patients (4.0 per 100 patient years vs. 2.1/100 patient years, respectively). Correa described the fall in risk of development of gastric cancer after removal of the major carcinogen (*H. pylori*) followed a sigmoid quadratic curve, and paralleled in reverse the progressive Correa pathway of gastric carcinogenesis [134].

## 12. Conclusions

Gastric carcinogenesis is a multifactorial and multistep process involving genetic susceptibility, environmental factors, *H. pylori* bacterial and *Epstein Barr* virus infection and epigenetics. Antioxidant systems and ingested phytochemicals can provide cytoprotection for the gastric epithelium against oxidative stress, ingested carcinogens, mutagens and chronic *Helicobacter pylori* infection. Ascorbic acid may be protective against gastric cancer through its antioxidant effect, by regenerating active vitamin E and glutathione, inhibiting endogenous N-nitrosation, reducing the somatic mutagenic effects of ingested nitrites, nitrosamines and heterocyclic amines, inhibiting damage from ROS and preventing *H. pylori* infection (or re-infection following eradication). Ascorbic acid is also an important co-factor in the demethylation of DNA suppressor genes.

It may be possible to prevent the progression of chronic atrophic gastritis and intestinal metaplasia to gastric cancer by *Helicobacter* eradication, risk reduction and gastric cytoprotection. The “point of no return” in *Helicobacter pylori* related gastric intestinal metaplasia may be important in the timing of *H. pylori* eradication programs in high risk groups. The effectiveness of gastric cytoprotection is closely related to the virulence of *H. pylori* strains, particularly CagA subtypes. Both intestinal and diffuse subtypes of gastric cancer share risk factors including *H. pylori* gastritis, blood group A, smoking and alchohol consumption. However, genetics (e.g., *CDH1* germline mutations) and female sex are important in diffuse gastric cancer. Recent transcriptomic and proteomic analysis has demonstrated that gastric cancer is a complex, heterogeneous disease, with substantial intra-tumoural, intra-patient and inter-patient variability. Eradication of *H. pylori* may lead to recovery of Vitamin C secretion by gastric epithelium and enable regression of premalignant gastric lesions, thereby interrupting the Correa pathway of gastric carcinogenesis.

Box 1 **Summary of Vitamin C actions in prevention of gastric carcinogenesis.**

- Scavenging of superoxide anion radical (O_2_^−^**·**), singlet oxygen (1O_2_), hydroxyl radical (OH**·**)
- Regeneration of vitamin E and glutathione
- Inhibition of lipid peroxidation in conjunction with catechins
- Enhanced activity of HIF-α Prolyl hydroxylase
- Enhanced activity of α-KGDD TET DNA hydroxylases
- Enhanced activity of JHDMs
- Decreased DNA CpG hypermethylation (CIMP)
- Upregulation of transmembrane protein with epidermal growth factor (EGF)-like and two follistatin motifs 2 (TMEFF2)
- Inhibition of endogenous gastric N-nitrosation
- Reduced toxicity of ingested heterocyclic amines/NDMA
- Inhibition of *H. pylori* urease
- Inhibition of *H. pylori* colonization
- Improved *H. pylori* eradication rates
- Inhibition of *H. pylori* mediated activation of NF-κB and STAT3
- Increased synthesis of PGE2 and gastric mucus production.

## Abbreviations

5-FU	5-fluorouracil
α-KGDDs	α-ketoglutarate-dependent dioxygenases
AG	Atrophic gastritis
AGE	Advanced glycation end product
AGS	Human gastric adenocarcinoma hyperdiploid cell line
*ALDH2*	Aldehyde Dehydrogenase 2 Family Member
ALE	Advanced lipid peroxidation end product
AlpA	Adherence associated lipoprotein A
Akt	Protein kinase B
APA	Anti-parietal cell antibodies
APC	Adenomatous polyposis coli protein
ATP	Adenosine triphosphate
BabA	Blood group antigen binding adhesin
BCL-2	B cell lymphoma-2
BRCA	Breast cancer susceptibility gene
CagA	Cytotoxin associated gene A
CAT	Catalase
CD	Cluster of differentiation
*CDH1*	E-cadherin gene
CDH1	E-cadherin protein
CDKN2A	Cyclin dependent Kinase Inhibitor 2A
CDX	Caudal type homeobox
CEACAM	Carcinoembryonic antigen-related cell adhesion molecule
CIMP	Cytosine and guanine (CpG) island methylator phenotype
CIN	Chromosome instability
COX	Cyclo-oxygenase
Csk	C-terminal Src kinase
CTGF	Connective tissue growth factor or CCN2
CXCL2	C-X-C chemokine ligand 2 or macrophage inflammatory protein 2-alpha (MIP)
CXCR4	C-X-C chemokine receptor type 4
DGC	Diffuse gastric cancer
DHA	Dehydroascorbic acid
dMMR	Deficient mismatch repair
EBV	*Epstein–Barr* virus
EGFR	Epidermal growth factor receptor
EMT	Epithelial mesenchymal transition
ENOC	Endogenous nitrosamine
EPIYA	Glutamate-proline isoleucinetyrosine-alanine
EPO	Erythropoietin
*erbB2*	Human epidermal growth factor receptor gene
ERK	Extracellular signal-regulated kinase
*etv4* ets	(Erythroblast Transformation Specific) variant transcription factor 4
FAK	Focal adhesion kinase
FAP	Familial adenomatous polyposis
*FGFR4*	Fibroblast growth factor receptor gene
GGT	γ-glutamyl transpeptidase
GLUT	Membrane glucose transporter
GPX	Glutathione peroxidase
GSH	Glutathione
GSTM1	Glutathione S-transferase Mu 1
GSTP1	Glutathione S-transferase Pi 1
GSTT1	Glutathione S-transferase Theta 1
HBD	Human β-defensin
HCA	Heterocyclic amine
HER2	Human epidermal growth factor
*HGF-R*	Hepatocyte growth factor receptor gene, c-MET
HIF-1α	Hypoxia inducible factor-1 alpha
HK	Hexokinase
HKalpha	Alpha subunit of the parietal cell proton pump
HMOX1	Heme oxygenase-1
hMLH1	Human MutL Homologue 1
HNO_2_	Nitrous acid
HNPCC	Hereditary non polyposis colorectal cancer
HOCl	Hypochlorous acid
HopQ	*Helicobacter pylori* outer protein Q
Hp NAP	*Helicobacter pylori* neutrophil-activating protein
HRE	Hypoxia response element
H_2_O_2_	Hydrogen peroxide
Htra	Serine protease high temperature requirement A
HSP	Heat shock protein
IFN	Interferon
IGFBP3	Insulin-like growth factor-binding protein 3
IκBα	Nuclear factor kappa light chain enhancer of activated B cells inhibitor, alpha
iNOS	Inducible nitric oxide synthase
IL	Interleukin
ILK	Integrin linked kinase
IM	Intestinal metaplasia
JHDM	Jumonji-C domain-containing histone demethylases
JNK	c-Jun N-terminal kinase
KRAS	Kirsten rat sarcoma virus oncogene
LDH	Lactate dehydrogenase
LOX	lysyl oxidase
L-GULO	L-gulono-gamma-lactone-oxidase
LPS	lipopolysaccharide
MALT	Mucosa associated lymphoid tissue
MAP3K	Mitogen-activating protein kinase
MDA	Malondialdehyde
MGMT	O-6-methylguanine DNA methyltransferase enzyme
MHC	Major histocompatibility complex
MMP	Matrix metalloprotease
MSI	Microsatellite instability
MSS	Microsatellite stable
mTOR	Mammalian target of rapamycin
NAC	N-acetyl cysteine
NDMA	Nitrosodimethylamine
NF-κB	Nuclear factor kappa light chain enhancer of activated B cells
NO	Nitric oxide
NOX	NADPH oxidase
OipA	Outer inflammatory protein A
PAMP	Pathogen associated molecular pattern
PDK1	Pyruvate dehydrogenase kinase 1
PDGFβ	Platelet derived growth factor beta
PD-L1	Programmed death-ligand 1
PGE2	Prostaglandin E2
PGK	Phosphoglycerate kinase
PGN	Peptidoglycan
PHD	Prolyl hydroxylase
PKM2	Pyruvate kinase muscle isoenzyme 2
PPI	Proton pump inhibitor
PTEN	Phosphatase and tensin homolog
PUFA	Polyunsaturated fatty acid
pVHL	Von-Hippel–Lindau protein
RAGE	Receptor for advanced glycation end product
RhoA	Ras homolog family member A
RICK	Receptor-interacting protein serine-threonine kinase 2
RNS	Reactive nitrogen species
ROS	Reactive oxygen species
SabA	Sialic acid binding adhesin
SFK	Src family kinase
SH2	Src Homology 2
SHIP2	Src Homology 2 inositol phosphatase
SIRT3	[sirtuin (silent mating type information regulation 2 homolog) 3]
SLC23A1	Solute carrier family 23 member 1
SLUG	Zinc finger protein SNAI2
SOD	Superoxide dismutase
SOX2	Sex determining region Y-box 2
SNAIL	Zinc finger protein SNAI1
SPEM	Spasmolytic polypeptide expressing metaplasia
Src	Non receptor associated sarcoma tyrosine kinase
STAT3	Signal transducer and activator of transcription 3
TET	Ten eleven translocation
TET1	TET methylcytosine dioxygenase 1
TF	Thompsen-Friedenreich antigen
T4SS	Type IV bacterial secretion system
*TGF-β*	Transforming growth factor beta gene
Th1	T helper type 1 cell
TLR	Toll-like receptor
Tlp	Transducer like protein
TNF-α	Tumor necrosis factor-alpha
TRAF	TNF Receptor associated factor
TRAIL	Tumour necrosis factor apoptosis inducing ligand
Tregs	Regulatory T cells
TWIST1	Twist basic helix loop helix transcription factor 1
vacA	Vacuolating cytotoxin gene A
VEGF	Vascular endothelial growth factor
VM	Vimentin
ZEB	Zinc finger E-box-binding homeobox 1
